# Different patterns of supra­molecular aggregation in three amides containing *N*-(benzo[*d*]thia­zol­yl) substituents

**DOI:** 10.1107/S2056989021003637

**Published:** 2021-04-09

**Authors:** Ninganayaka Mahesha, Hemmige S. Yathirajan, Holalagudu A. Nagma Banu, Balakrishna Kalluraya, Sabine Foro, Christopher Glidewell

**Affiliations:** a Department of Studies in Chemistry, University of Mysore, Manasagangotri, Mysuru-570 006, India; b Department of Studies in Chemistry, Mangalore University, Mangalagangotri, Mangalore-574199, India; c Institute of Materials Science, Darmstadt University of Technology, Alarich-Weiss-Strasse 2, D-64287 Darmstadt, Germany; d School of Chemistry, University of St Andrews, St Andrews, Fife KY16 9ST, UK

**Keywords:** heterocyclic compounds, benzo[*d*]thia­zoles, crystal structure, mol­ecular conformation, disorder, hydrogen bonding, halogen bonding

## Abstract

Crystal structures are reported for three amides containing *N*-benzo[*d*]thia­zole substituents. In *N*-(benzo[*d*]thia­zol-6-yl)-3-bromo­benzamide, C_14_H_9_BrN_2_OS, where the two ring systems are nearly parallel to one another [dihedral angle = 5.8 (2)°], the mol­ecules are linked by N—H⋯O and C—H⋯N hydrogen bonds to form ribbons of *R* ^3^_3_(19) rings, which are linked into sheets by short Br⋯Br inter­actions [3.5812 (6) Å]. *N*-(6-Meth­oxy­benzo[*d*]thia­zol-2-yl)-2-nitro­benzamide, C_15_H_11_N_3_O_4_S, crystallizes with *Z*′ = 2 in space group *Pna*2_1_: the dihedral angles between the ring systems [46.43 (15) and 66.35 (13)°] are significantly different in the independent mol­ecules and a combination of two N—H⋯N and five C—H⋯O hydrogen bonds links the mol­ecules into a three-dimensional network. The mol­ecules of 5-cyclo­propyl-*N*-(6-meth­oxy­ben­zo[*d*]thia­zol-2-yl)­isoxazole-3-carboxamide, C_15_H_13_N_3_O_3_S, exhibit two forms of disorder, in the meth­oxy group and in the cyclo­propyl­isoxazole unit; symmetry-related pairs of mol­ecules are linked into dimers by pairwise N—H⋯N hydrogen bonds. Comparisons are made with the structures of some related compounds.

## Abstract 2

In three amides, each containing a *N*-(benzo[*d*]thia­zol­yl) substituent, different combinations of N—H⋯O, N—H⋯N, C—H⋯O and C—H⋯N hydrogen bonds and Br⋯Br inter­actions lead to supra­molecular assemblies in one, two and three dimensions.

## Chemical context   

Compounds containing the benzo[*d*]thia­zole unit exhibit a wide range of biological and medicinal activities, which have been reviewed by Henary *et al.* (2013 ▸). Notable examples include the presence of the benzo[*d*]thia­zole nucleus in firefly luciferin, (4*S*)-2-(6-hy­droxy­benzo[*d*]thia­zol-2-yl)-4,5-di­hydro­thia­zole-4-carb­oxy­lic acid (White *et al.*, 1963 ▸), action as potent and selective human adenosine A_3_ receptor antagonists (Jung *et al.*, 2004 ▸) and cholinesterase inhibitors (Imramovský *et al.*, 2013 ▸). In addition, applications in Green Chemistry have very recently been reviewed (Gao *et al.*, 2020 ▸).

Against this diverse background, we report here the synthesis and structures of three carboxamides containing the benzo[*d*]thia­zole nucleus, namely: *N*-(benzo[*d*]thia­zol-6-yl)-3-bromo­benzamide (I), *N*-(6-meth­oxy­benzo[*d*]thia­zol-2-yl)-2-nitro­benzamide (II) and *N*-(6-meth­oxy­benzo[*d*]thia­zol-2-yl)-5-cyclo­propyl­isoxazole-3-carboxamide (III). Compounds (I)–(III) were prepared in yields exceeding 85% by the reaction of an amino-substituted benzo[*d*]thia­zole with an acid chloride in the presence of tri­ethyl­amine.
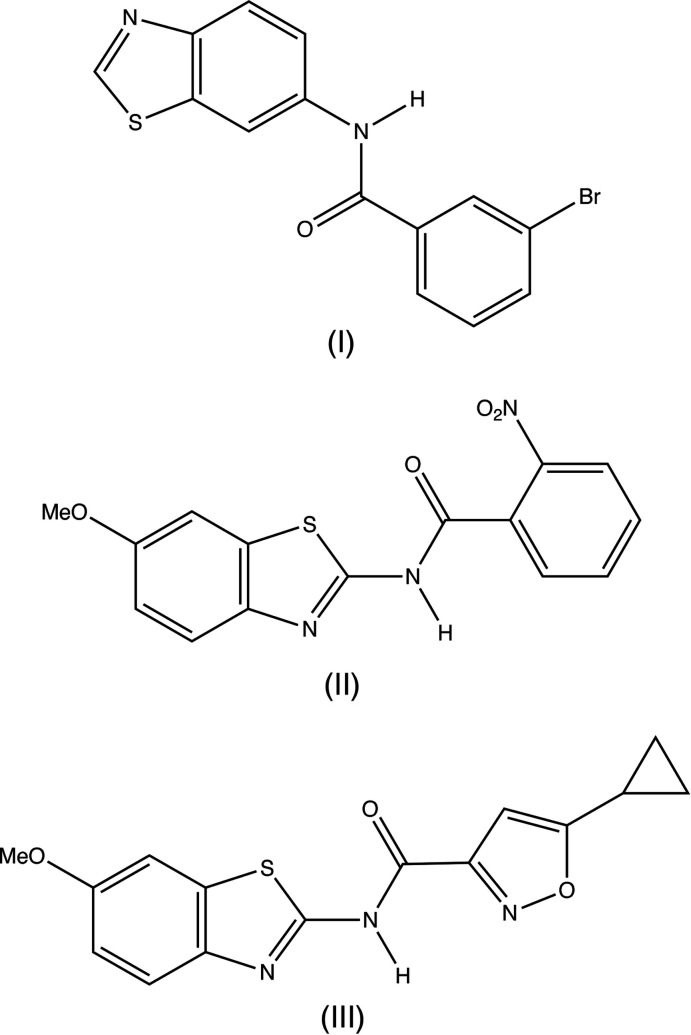


## Structural commentary   

**Figure 1:**
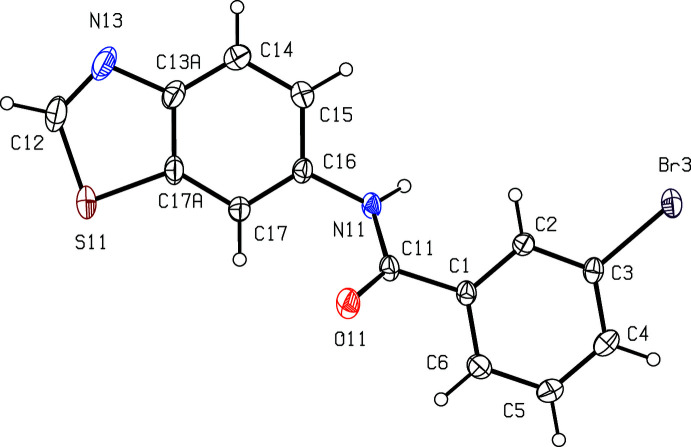
The mol­ecular structure of (I), showing the atom-labelling scheme. Displacement ellipsoids are drawn at the 30% probability level.

In compound (I), the amide unit occupies position 6 of the benzo[*d*]thia­zole unit, whereas in compounds (II) and (III), the amide unit is linked to the bicyclic system at position 2. In (I), (Fig. 1 ▸) the thia­zole ring and the brominated aryl ring are almost parallel, with a dihedral angle between them of 5.8 (2)°. However, these rings are not coplanar, as both ring systems in compound (I) are twisted out of the plane of the central amide spacer unit.

**Figure 2:**
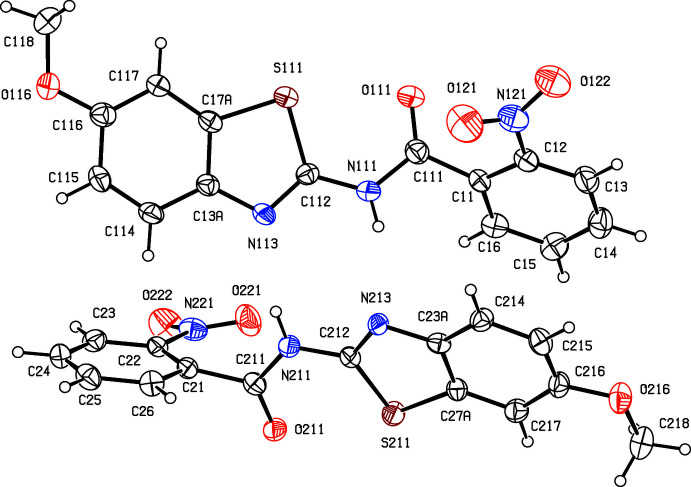
The structures of the two independent mol­ecules in (II), showing the atom-labelling scheme. Displacement ellipsoids are drawn at the 30% probability level.

Compound (II) crystallizes with *Z*′ = 2, but a search for possible additional crystallographic symmetry revealed none. The different conformations of the two independent mol­ecules (Fig. 2 ▸) confirm the absence of additional symmetry. For example, the dihedral angle between the thia­zole ring and the nitrated phenyl ring is 46.43 (15)° in mol­ecule 1 containing atom S111, but 66.35 (13)° in mol­ecule 2 containing atom S211. Similarly, the dihedral angles between the nitro groups and the adjacent aryl rings are 34.5 (2) and 17.9 (2)° in mol­ecules 1 and 2, respectively.

**Figure 3:**
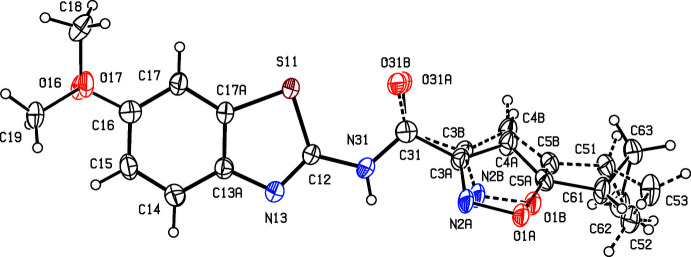
The mol­ecular structure of (III), showing the atom-labelling scheme and the disorder of the cyclo­propyl­isoxazole fragment, where the major disorder component, with occupancy 0.549 (5), is drawn using full lines and the minor disorder component of this fragment, with occupancy 0.451 (5), is drawn using broken lines. The atomic sites O16, O17, C18 and C19 and the associated H atoms all have occupancy 0.5 (see Section 2). Displacement ellipsoids are drawn at the 30% probability level.

The mol­ecule of compound (III) exhibits two forms of disorder. The cyclo­propyl­isoxazole unit is disordered over two sets of atomic sites, with occupancies 0.549 (5) and 0.451 (5), where the two orientations of the isoxazole ring are approximately related by small rotations about the N—C and C—C bonds involving atom C31 (Fig. 3 ▸). Of more inter­est is the disorder of the meth­oxy groups, where the site occupancies are constrained by short non-bonded contacts with adjacent mol­ecules. Thus, the atomic site C18 in the mol­ecule at (*x*, *y*, *z*) is only 1.840 (8) Å from the corresponding site in the mol­ecule at (2 − *x*, *y*, 1.5 − *z*): hence, only one of these sites can be occupied and this, in turn, limits this site occupancy in each mol­ecule to a maximum value of 0.5. Similarly, the atomic site C19 at (*x*, *y*, *z*) is only 1.921 (9) Å from the corresponding site in the mol­ecule at (2 − *x*, 1 − *y*, 1 − *z*), again limiting the site occupancy to a maximum value of 0.5. Hence the site occupancy for each orientation of the meth­oxy group must each be exactly 0.5.

In each of the independent meth­oxy groups in compound (II), and for each orientation of the meth­oxy group in compound (III), the two exocyclic C—C—O angles differ by *ca* 10%, as is generally found in planar, or nearly planar, alk­oxy­arenes (Seip & Seip, 1973 ▸; Ferguson *et al.*, 1996 ▸). In compounds (II) and (III), the maximum displacement of any meth­oxy C atoms from the plane of the adjacent aryl ring is 0.144 (9) Å for atom C218 in compound (II).

## Supra­molecular features   

**Figure 4:**
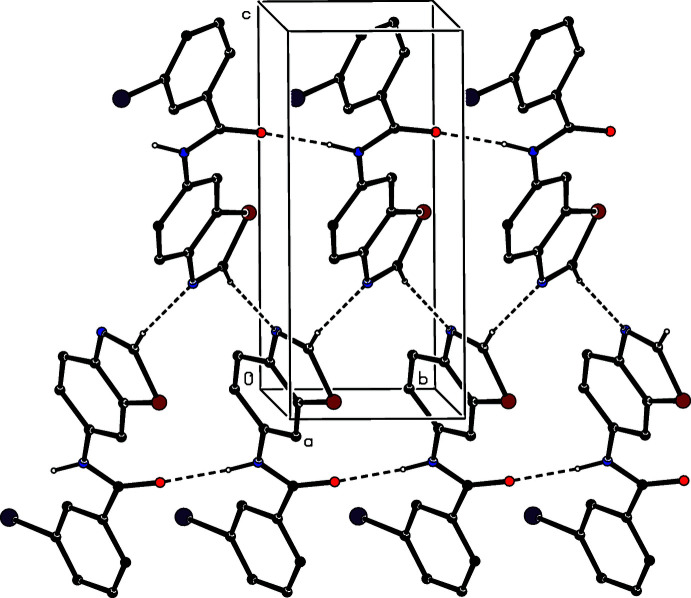
Part of the crystal structure of (I) showing the formation of a ribbon of 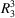
(19) rings running parallel to [010] and built from N—H⋯O and C—H⋯N hydrogen bonds. Hydrogen bonds are drawn as dashed lines and, for the sake of clarity, the H atoms not involved in the motifs shown have been omitted.

**Figure 5:**
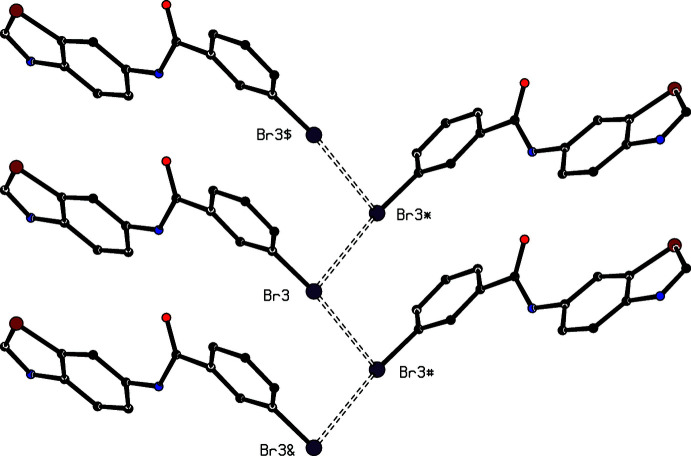
Part of the crystal structure of (I), showing a chain along ([010] containing two independent Br⋯Br inter­actions (shown as dashed lines). For the sake of clarity, the H atoms and the unit-cell outline have been omitted. The Br atoms marked with an asterisk (*), a hash (#), a dollar sign ($) or an ampersand (&) are at the symmetry positions (2 − *x*, 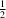 + *y*, 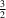 − *z*), (2 − *x*, −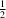 + *y*, 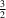 − *z*), (*x*, 1 + *y*, *z*) and (*x*, −1 + *y*, *z*), respectively.

**Figure 6:**
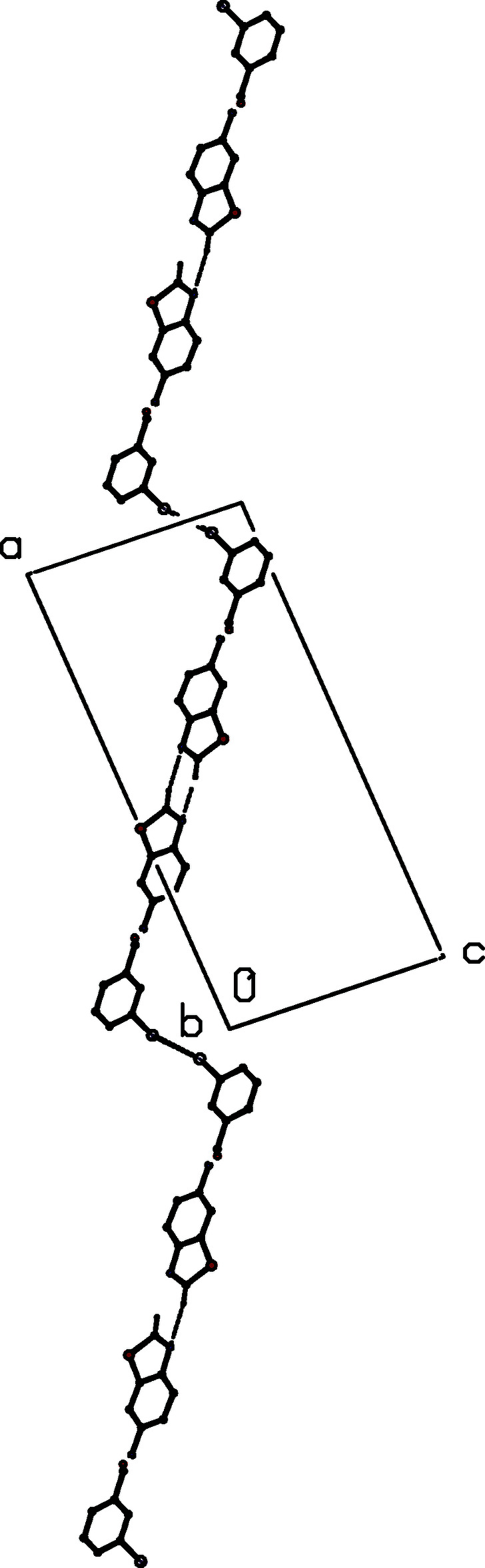
A projection along [010] of part of the crystal structure of (I) showing how the Br⋯Br inter­actions (dashed lines) link the hydrogen-bonded ribbons into sheets lying parallel to (10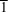).

The supra­molecular assembly of compound (I) is built up from N—H⋯O and C—H⋯N hydrogen bonds (Table 1 ▸). Mol­ecules related by translation are linked by N—H⋯O hydrogen bonds to form a *C*(4) (Etter, 1990 ▸; Etter *et al.*, 1990 ▸; Bernstein *et al.*, 1995 ▸) chain, of the type very commonly found in simple amides (Fun *et al.*, 2011*a* ▸,*b* ▸; Praveen *et al.*, 2011 ▸; Fun, Quah *et al.*, 2012 ▸; Fun, Shahani *et al.*, 2012 ▸; Praveen *et al.*, 2013*a* ▸,*b* ▸; Nayak *et al.*, 2014 ▸): in (I), this chain runs parallel to the [010] direction (Fig. 4 ▸). In addition, mol­ecules that are related by the 2_1_ screw axis along (0.5, *y*, 0.25) are linked by C—H⋯N hydrogen bonds to form a *C*(6) chain, also running parallel to the [010] direction. The combination of these two chain motifs generates a ribbon of 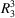
(19) rings along [010] (Fig. 4 ▸). Also running through the unit cell is a second ribbon of this type, related to the first by inversion, and containing mol­ecules that are related by the 2_1_ screw axis along (0.5, *y*, 0.75). Also present in the structure of compound (I) are two inter­molecular Br⋯Br contacts that are shorter than the van der Waals radii sum of 3.74 Å (Rowland & Taylor, 1996 ▸). Atom Br3 in the mol­ecule at (*x*, *y*, *z*) makes contacts with the corresponding atoms at (2 − *x*, 0.5 + *y*, 1.5 − *z*) and (2 − *x*, −0.5 + *y*, 1.5 − *z*), with Br⋯Br distances of 3.5812 (6) Å in each case; however, the C—Br⋯Br angles are 92.64 (18) and 166.44 (10)°, respectively (Fig. 5 ▸), which are consistent with the angular preferences found for such contacts from database analyses (Ramasubbu *et al.*, 1986 ▸). The effects of these halogen bonds (Cavallo *et al.*, 2016 ▸) are twofold: firstly to generate a chain running parallel to the [010] direction (Fig. 5 ▸) and thence to link the hydrogen-bonded ribbons into sheets lying parallel to the (10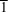) plane (Fig. 6 ▸).

**Figure 7:**
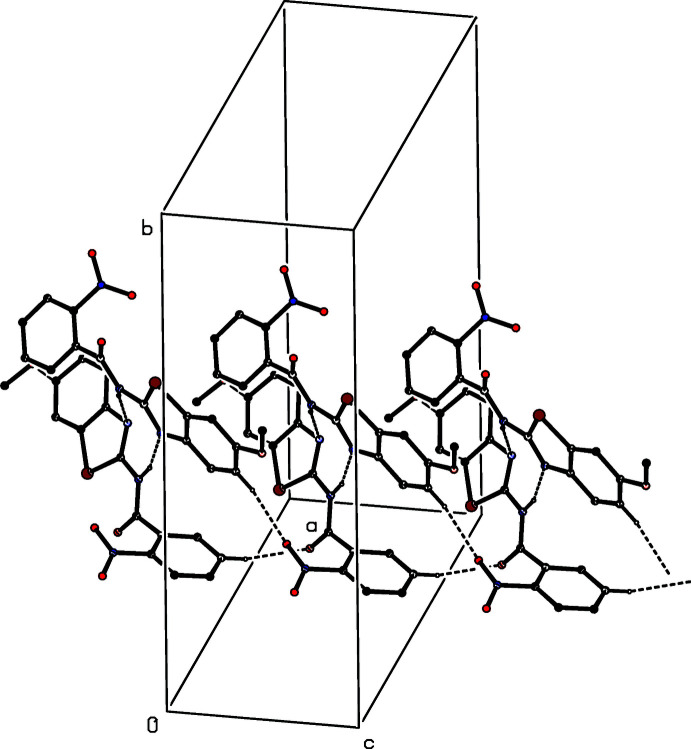
Part of the crystal structure of (II) showing the formation of a chain of 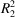
(8) and 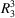
(18) rings running parallel to [001] and built from N—H⋯N and C—H⋯O hydrogen bonds. Hydrogen bonds are drawn as dashed lines and, for the sake of clarity, the H atoms not involved in the motifs shown have been omitted.

**Figure 8:**
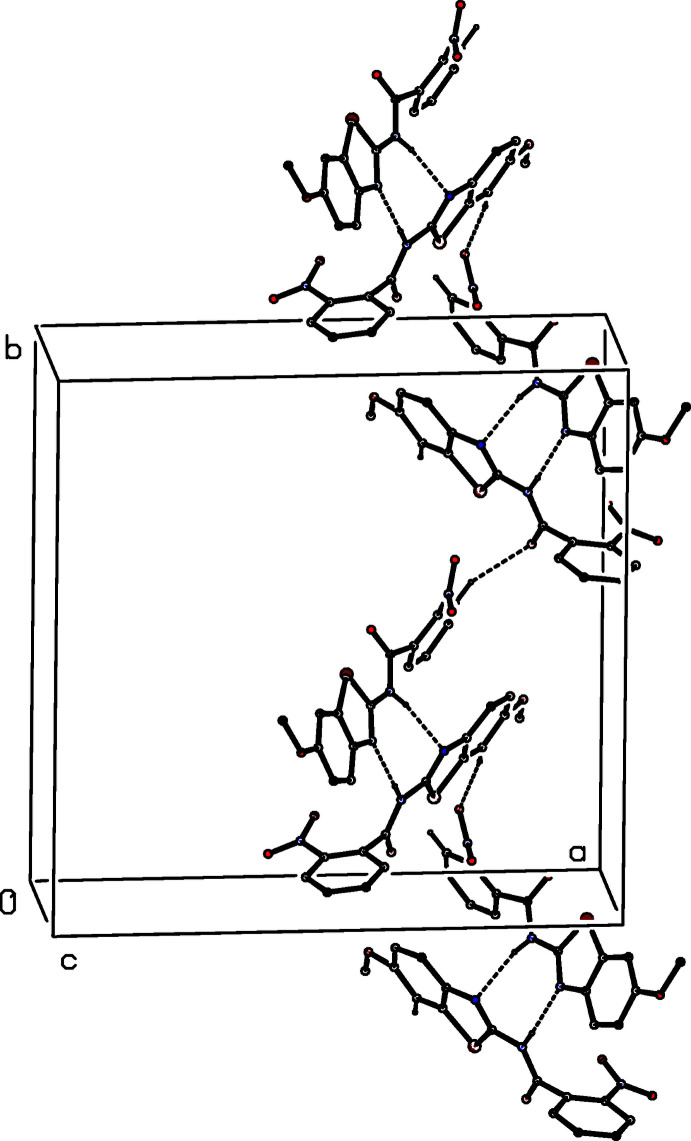
Part of the crystal structure of (II) showing the formation of a chain of rings running parallel to [010] and built from N—H⋯N and C—H⋯O hydrogen bonds. Hydrogen bonds are drawn as dashed lines and, for the sake of clarity, the H atoms not involved in the motifs shown have been omitted.

**Figure 9:**
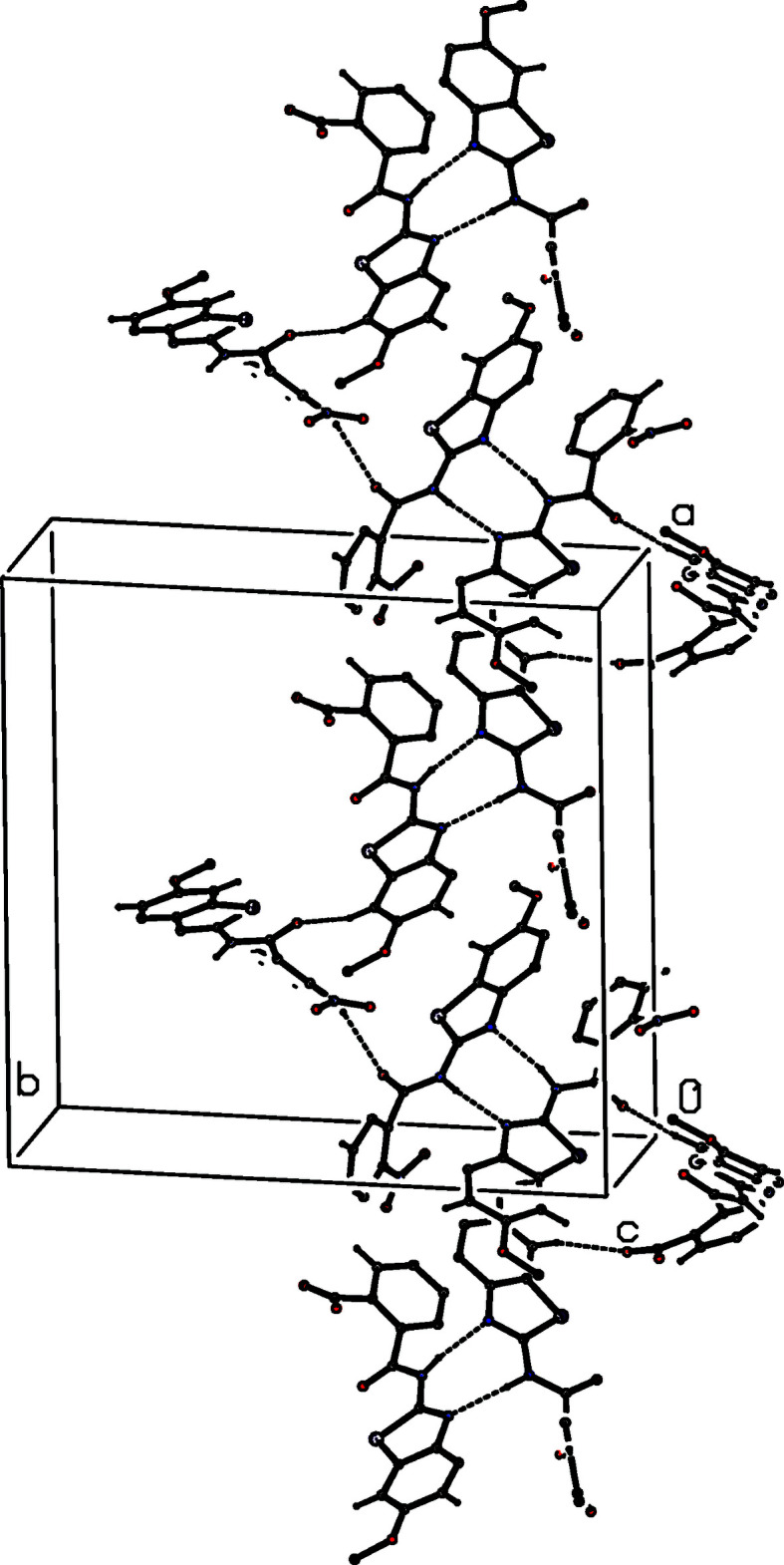
Part of the crystal structure of (II) showing the formation of a chain of rings running parallel to [100] and built from N—H⋯N and C—H⋯O hydrogen bonds. Hydrogen bonds are drawn as dashed lines and, for the sake of clarity, the H atoms not involved in the motifs shown have been omitted.

The two independent mol­ecules of compound (II) are linked by two N—H⋯N hydrogen bonds and five C—H⋯O hydrogen bonds (Table 1 ▸), but the N—H⋯O hydrogen bonds typical of amides are absent. The hydrogen bonds generate a three-dimensional network, whose formation can readily be analysed in terms of a number of simple sub-structures (Ferguson *et al.*, 1998*a* ▸,*b* ▸; Gregson *et al.*, 2000 ▸). In the simplest of the sub-structures, the two N—H⋯N hydrogen bonds link the mol­ecules within the selected asymmetric unit to form a dimer, and the other sub-structures follow the different ways in which these dimers can be linked. The C—H⋯O hydrogen bonds involving atoms C25 and C115 link the dimers into a chain of alternating 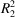
(8) 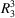
(18) rings running parallel to the [001] direction (Fig. 7 ▸); this chain is weakly reinforced by a C—H⋯π(arene) inter­action (Table 1 ▸). In the third sub-structure, the C—H⋯O hydrogen bonds involving atoms C13 and C217 link the dimers into a chain of rings containing 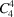
(24) chains and running parallel to the [010] direction (Fig. 8 ▸). The combination of the chains along [010] and [001] generates a sheet lying parallel to (100) in the domain 0.5 < *x* < 1.0. A second sheet of the type, related to the first by the 2_1_ screw axes, lies in the domain 0 < *x* < 0.5, and sheets of this type are linked by the C—H⋯O hydrogen bond in involving atom C117, so forming a three-dimensional network: indeed, it is possible to identify a complex chain running parallel to the [100] direction, which defines the linkage of the (100) sheets (Fig. 9 ▸).

**Figure 10:**
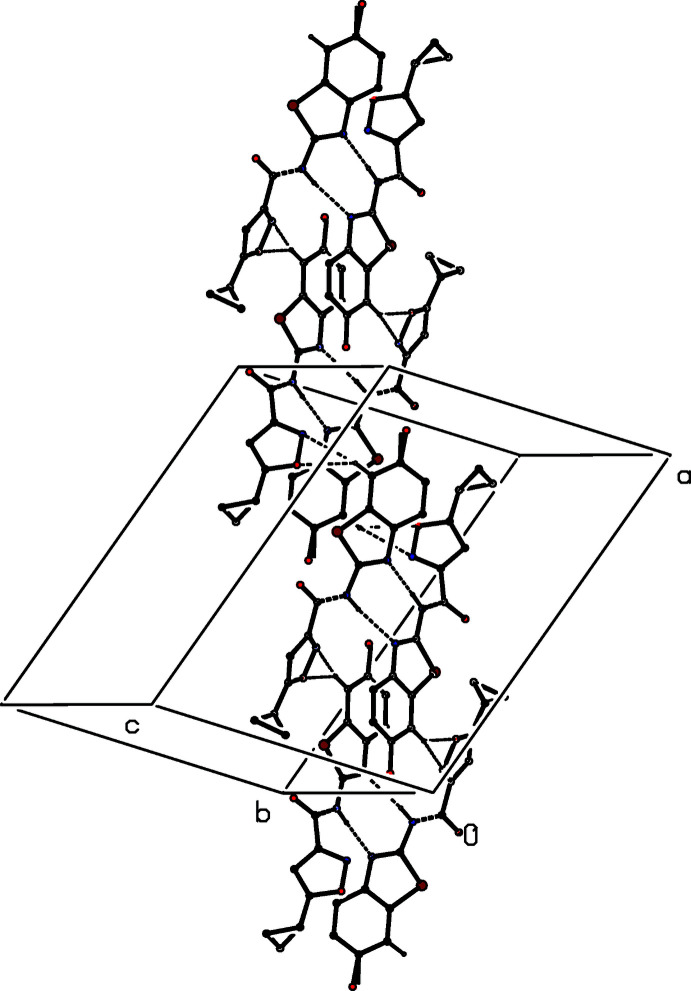
Part of the crystal structure of (III) showing the formation of a chain of rings running parallel to the [101] direction. For the sake of clarity, the methyl groups, the minor disorder component and the H atoms which are not involved in the motif shown have all been omitted.

Analysis of the supra­molecular aggregation in compound (III) is complicated by the disorder of the isoxazole ring, since atoms O1*A* and N2*A* in the major disorder form act as hydrogen bond acceptors, but atoms O1*B* and N2*B* in the minor disorder form do not. As in (II), the N—H⋯O hydrogen bonds typical of amides are absent from the structure of (III). Mol­ecules of (III) that are related by a twofold rotation axis are linked into cyclic 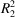
(8) dimers. There is also present an asymmetric three-centre C—H⋯(N,O) system having atoms O1*A* and N2*A* as the acceptors: if these sites had full occupancy, this inter­action would generate a chain of rings running parallel to the [101] direction (Fig. 10 ▸). However, because of the disorder, this chain is punctuated rather than continuous.

## Database survey   

*N*-(Benzo[*d*]thia­zol-2-yl)-3-bromo­benzamide (IV) [CSD (Groom *et al.*, 2016 ▸) refcode SUQTAC; Odame *et al.*, 2020 ▸] is a positional isomer of compound (I), with the amide substituent as position 2 of the benzo­thia­zole unit, rather than at position 6 as in (I). In contrast to compound (I), but consistent with compounds (II) and (III), where the amide units are also linked to the heterocycle at position 6, the structure of (IV) contains no N—H⋯O hydrogen bonds: instead, inversion-related pairs of mol­ecules are linked by pairwise N—H⋯N hydrogen bonds to form cyclic, centrosymmetric 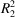
(8) dimers. By contrast with (I), there are no short Br⋯Br contacts in the structure of (IV).

In the simple amine 2-amino-6-methyl­benzo[*d*]thia­zole, which crystallizes with *Z*′ = 2 in space group *P* 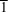 (GINBIP; Saeed *et al.*, 2007 ▸), the mol­ecules are linked into complex chains by a combination of three N—H⋯N hydrogen bonds and one N—H⋯π(arene) hydrogen bond, while in the closely related 2-amino-6-nitro­benzo[*d*]thia­zole (TIJLUT; Glidewell *et al.*, 2001 ▸), inversion-related mol­ecules are once again linked by pairwise N—H⋯N hydrogen bonds to form 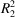
(8) dimers, which are further linked by a three-centre N—H⋯(O,O) system to form a three-dimensional network.

## Synthesis and crystallization   

All reagents were obtained commercially and all were used as received. For the synthesis of compound (I), a solution of tri­ethyl­amine (1.11 g, 0.01 mol) in dry toluene (5 ml) was added to a mixture of 6-amino­benzo[*d*]thia­zole (1.50 g, 0.01 mol) and 3-bromo­benzoyl chloride (2.18 g, 0.01 mol) in dry toluene (20 ml), and the resulting mixture was heated under reflux for 4 h. When the reaction was complete, as indicated by TLC monitoring, the mixture was cooled to room temperature and the tri­ethyl­ammonium chloride was removed by filtration. The solvent was then removed under reduced pressure and the resulting solid product was washed with water and then crystallized from ethanol solution. Yield 86%, m.p. 439–441 K: IR (cm^−1^) 3125 (N—H), 1667 (C=O), 1616 (C=N); NMR (CDCl_3_) δ(^1^H) 7.90 (*s*, 1H, thia­zole), 8.21 (*s*, 1H), NH), 6.8–7.9 (*m*, 7H, aromatic); MS (70 eV) *m*/*z* 335/333, relative intensities 1:1 (*M* ^+^ + 1). Compound (II) was prepared in a similar manner, using 2-amino-6-meth­oxy­benzo[*d*]thia­zole (1.80 g, 0.01 mol) and 2-nitro­benzoyl chloride (1.85 g, 0.01 mol). Yield 87%, m.p. 468–470 K; IR (cm^−1^) 3150 (N—H), 1681 (C=O), 1615 (C=N), 1560 and 1346 (nitro); NMR (CDCl_3_) δ(^1^H) 3.80 (*s*, 3H, OMe), 7.2–8.6 (*m*, 7H, aromatic), 8.10 (*s*, 1H, NH); MS (70 eV) *m*/*z* 330 (*M* ^+^ + 1). Compound (III) was similarly prepared using 2-amino-6-meth­oxy­benzo[*d*]thia­zole (1.80 g, 0.01 mol) and 5-cyclo­propyl­isoxazole-3-carboxyl­chloride (1.71 g, 0.01mol). Yield 88%, m.p. 453 K: IR (cm^−1^) 3120 (N—H), 1676 (C=O), 1625 (C=N); NMR (DMSO-*d*_6_) δ(^1^H) 0.2–2.1 (*m*, 5H, cyclo­prop­yl), 3.83 (*s*, 3H, OMe), 6.90 (*s*, 1H, H-17), 7.20 (*d*, 1H, *J* = 7.4 Hz) and 7.46 (*d*, 1H, *J* = 7.4 Hz) ((H-14 and H-15), 7.80 (*s*, 1H, H-4); MS (70 eV) *m*/*z* 316 (*M* ^+^ + 1).

## Refinement   

Crystal data, data collection and structure refinement details are summarized in Table 2 ▸. One bad outlier reflection (0,23,3) was omitted from the final refinement of compound (II). All H atoms, apart from those in the disordered components of compound (III), were located in difference maps. The H atoms bonded to C atoms were treated as riding atoms in geometrically idealized positions, with C—H distances of 0.93 Å (aromatic and heterocyclic), 0.96 Å (CH_3_), 0.97 Å (CH_2_) or 0.98 Å (aliphatic C—H) and with *U*_iso_(H) = *kU*_eq_(C), where *k* = 1.5 for the methyl groups, which were allowed to rotate but not to tilt, and 1.2 for all other H atoms bonded to C atoms. For the H atoms bonded to N atoms, the atomic coordinates were refined with *U*_iso_(H) = 1.2*U*_eq_(N), giving the N—H distances shown in Table 1 ▸. For the disordered methyl group in compound N3, the site occupancies were fixed at 0.5 (see Section 2, above): when these occupancies were refined, the resulting values were 0.504 (7) and 0.496 (7), much as expected. For each of the disordered fragments in (III), the corresponding bonded distances and the 1,3 non-bonded distances were restrained to be equal, subject to s.u. values of 0.01 and 0.02 Å, respectively. In addition, the anisotropic displacement parameters for corresponding pairs of atoms in the 3-cyclo­propyl-5-carbonyl­oxazole fragments were constrained to be equal. Subject to these conditions, the occupancies of this disordered fragment refined to 0.549 (5) and 0.451 (5). The correct orientation of the structure of the crystal of compound (II) chosen for data collection relative to the polar axis direction was established by means of the Flack *x* parameter (Flack, 1983 ▸); *x* = 0.02 (5), calculated (Parsons *et al.*, 2013 ▸) using 708 quotients of the type [(*I* ^+^) − (*I* ^−^)]/[(*I* ^+^) + (*I* ^−^)].

## Supplementary Material

Crystal structure: contains datablock(s) global, I, II, III. DOI: 10.1107/S2056989021003637/hb7974sup1.cif

Structure factors: contains datablock(s) I. DOI: 10.1107/S2056989021003637/hb7974Isup2.hkl

Structure factors: contains datablock(s) II. DOI: 10.1107/S2056989021003637/hb7974IIsup3.hkl

Structure factors: contains datablock(s) III. DOI: 10.1107/S2056989021003637/hb7974IIIsup4.hkl

Supporting information file. DOI: 10.1107/S2056989021003637/hb7974Isup5.cml

Supporting information file. DOI: 10.1107/S2056989021003637/hb7974IIsup6.cml

Supporting information file. DOI: 10.1107/S2056989021003637/hb7974IIIsup7.cml

CCDC references: 2075271, 2075270, 2075269

Additional supporting information:  crystallographic information; 3D view; checkCIF report

## Figures and Tables

**Table 1:** Hydrogen bonds and short inter­molecular contacts(Å, °) *Cg*1 represents the centroid of the ring C13*A*/C17*A*/C117/C116/C115/C114

Compound	*D*—H⋯*A*	*D*—H	H⋯*A*	*D*⋯*A*	*D*—H⋯*A*
(I)	N11—H11⋯O11^i^	0.90 (4)	1.97 (3)	2.840 (4)	164 (3))
	C12—H12⋯N13^ii^	0.93	2.62	3.512 (6)	161
					
(II)	N111—H111⋯N213	0.82 (4)	2.19 (4)	2.981 (5)	165 (4)
	N211—H211⋯N113	0.86 (4)	2.17 (4)	2.992 (5)	162 (4)
	C13—H13⋯O211^iii^	0.93	2.53	3.408 (7)	158
	C25—H25⋯O211^iv^	0.93	2.44	3.349 (6)	165
	C115—H115⋯O221^iv^	0.93	2.45	3.353 (7)	163
	C117—H117⋯O111^v^	0.93	2.44	3.236 (5)	144
	C217—H217⋯O122^vi^	0.93	2.51	3.412 (6)	164
	C16—H16⋯*Cg*1^vii^	0.93	2.84	3.484 (6)	128
					
(III)	N31—H31⋯N13^viii^	0.82 (3)	2.19 (3)	3.003 (3)	173 (2)
	C17—H17⋯O1*A* ^ix^	0.93	2.51	3.293 (7)	142
	C17—H17⋯N2*A* ^ix^	0.93	2.55	3.440 (19)	160
	C63—H63*B*⋯O31*A* ^*x*^	0.97	2.58	3.440 (18)	148

Symmetry codes: (i) *x*, −1 + *y*, *z*; (ii) 1 − *x*, {1\over 2} + *y*, {1\over 2} − *z*; (iii) {3\over 2} − *x*, {1\over 2} + *y*, −{1\over 2} − *z*; (iv) *x*, *y*, 1 + *z*; (v) 1 − *x*, 1 − *y*, {1\over 2} + *z*; (vi) {3\over 2} − *x*, −{1\over 2} + *y*, −{1\over 2} − *z*; (vii) *x*, *y*, −1 + *z*; (viii) 1 − *x*, *y*, {1\over 2} − *z*; (ix) {1\over 2} + *x*, {3\over 2} − *y*, {1\over 2} + *z*; (*x*) {1\over 2} − *x*, {3\over 2} − *y*, 1 − *z*.

**Table 2:** Experimental details

	(I)	(II)	(III)
Crystal data			
Chemical formula	C_14_H_9_BrN_2_OS	C_15_H_11_N_3_O_4_S	C_15_H_13_N_3_O_3_S
*M*_r_	333.19	329.33	315.34
Crystal system, space group	Monoclinic, *P*2_1_/*c*	Orthorhombic, *P* *n* *a*2_1_	Monoclinic, *C*2/*c*
Temperature (K)	296	296	296
*a*, *b*, *c* (Å)	24.221 (1), 4.9481 (3), 10.9981 (6)	20.085 (2), 20.165 (2), 7.3220 (6)	18.720 (1), 11.5255 (8), 14.7905 (9)
α, β, γ (°)	90, 95.371 (5), 90	90, 90, 90	90, 115.52 (1), 90
*V* (Å^3^)	1312.31 (12)	2965.5 (5)	2879.8 (4)
*Z*	4	8	8
Radiation type	Mo *K*α	Mo *K*α	Mo *K*α
μ (mm^−1^)	3.28	0.24	0.24
Crystal size (mm)	0.50 × 0.36 × 0.08	0.50 × 0.12 × 0.10	0.30 × 0.20 × 0.10

Data collection			
Diffractometer	Oxford Diffraction Xcalibur CCD	Oxford Diffraction Xcalibur CCD	Oxford Diffraction Xcalibur CCD
Absorption correction	Multi-scan (*CrysAlis RED*; Oxford Diffraction, 2009 ▸)	Multi-scan (*CrysAlis RED*; Oxford Diffraction, 2009 ▸)	Multi-scan (*CrysAlis RED*; Oxford Diffraction, 2009 ▸)
*T*_min_, *T*_max_	0.168, 0.769	0.787, 0.976	0.908, 0.976
No. of measured, independent and observed [*I* > 2σ(*I*)] reflections	4839, 2805, 2170	8012, 4784, 2969	5937, 3118, 1730
*R*_int_	0.031	0.040	0.038
(sin θ/λ)_max_ (Å^−1^)	0.656	0.661	0.656

Refinement			
*R*[*F* ^2^ > 2σ(*F* ^2^)], *wR*(*F* ^2^), *S*	0.043, 0.123, 1.03	0.049, 0.075, 1.05	0.056, 0.119, 1.02
No. of reflections	2805	4784	3118
No. of parameters	175	423	268
No. of restraints	0	1	26
H-atom treatment	H atoms treated by a mixture of independent and constrained refinement	H atoms treated by a mixture of independent and constrained refinement	H atoms treated by a mixture of independent and constrained refinement
Δρ_max_, Δρ_min_ (e Å^−3^)	1.00, −0.73	0.19, −0.22	0.23, −0.24
Absolute structure	–	Flack x parameter (Parsons *et al.*, 2013 ▸)	–
Absolute structure parameter	–	0.02 (5)	–

Computer programs: *CrysAlis CCD* and *CrysAlis RED* (Oxford Diffraction, 2009 ▸), *SHELXT* (Sheldrick, 2015*a* ▸), *SHELXL2014* (Sheldrick, 2015*b* ▸) and *PLATON* (Spek, 2020 ▸).

## supplementary crystallographic information

### N-(Benzo[d]thiazol-6-yl)-3-bromobenzamide (I) Crystal data

**Table:**

C_14_H_9_BrN_2_OS	*F*(000) = 664
*M**_r_* = 333.19	*D*_x_ = 1.686 Mg m^−^^3^
Monoclinic, *P*2_1_/*c*	Mo *K*α radiation, λ = 0.71073 Å
*a* = 24.221 (1) Å	Cell parameters from 2805 reflections
*b* = 4.9481 (3) Å	θ = 2.5–27.8°
*c* = 10.9981 (6) Å	µ = 3.28 mm^−^^1^
β = 95.371 (5)°	*T* = 296 K
*V* = 1312.31 (12) Å^3^	Plate, colourless
*Z* = 4	0.50 × 0.36 × 0.08 mm

### N-(Benzo[d]thiazol-6-yl)-3-bromobenzamide (I) Data collection

**Table:**

Oxford Diffraction Xcalibur CCD diffractometer	2805 independent reflections
Radiation source: Enhance (Mo) X-ray Source	2170 reflections with *I* > 2σ(*I*)
Graphite monochromator	*R*_int_ = 0.031
ω scans	θ_max_ = 27.8°, θ_min_ = 2.5°
Absorption correction: multi-scan (CrysalisRed; Oxford Diffraction, 2009)	*h* = −31→24
*T*_min_ = 0.168, *T*_max_ = 0.769	*k* = −4→6
4839 measured reflections	*l* = −10→14

### N-(Benzo[d]thiazol-6-yl)-3-bromobenzamide (I) Refinement

**Table:**

Refinement on *F*^2^	Primary atom site location: difference Fourier map
Least-squares matrix: full	Hydrogen site location: mixed
*R*[*F*^2^ > 2σ(*F*^2^)] = 0.043	H atoms treated by a mixture of independent and constrained refinement
*wR*(*F*^2^) = 0.123	*w* = 1/[σ^2^(*F*_o_^2^) + (0.0826*P*)^2^] where *P* = (*F*_o_^2^ + 2*F*_c_^2^)/3
*S* = 1.03	(Δ/σ)_max_ = 0.001
2805 reflections	Δρ_max_ = 1.00 e Å^−^^3^
175 parameters	Δρ_min_ = −0.73 e Å^−^^3^
0 restraints	

### N-(Benzo[d]thiazol-6-yl)-3-bromobenzamide (I) Special details

**Table:**

Geometry. All esds (except the esd in the dihedral angle between two l.s. planes) are estimated using the full covariance matrix. The cell esds are taken into account individually in the estimation of esds in distances, angles and torsion angles; correlations between esds in cell parameters are only used when they are defined by crystal symmetry. An approximate (isotropic) treatment of cell esds is used for estimating esds involving l.s. planes.

### N-(Benzo[d]thiazol-6-yl)-3-bromobenzamide (I) Fractional atomic coordinates and isotropic or equivalent isotropic displacement parameters (Å^2^)

**Table:**

	*x*	*y*	*z*	*U*_iso_*/*U*_eq_	
C1	0.82689 (14)	0.5633 (6)	0.8202 (3)	0.0285 (7)	
C2	0.86414 (13)	0.3673 (6)	0.7894 (3)	0.0292 (7)	
H2	0.8588	0.2773	0.7150	0.035*	
C3	0.90946 (13)	0.3081 (7)	0.8714 (3)	0.0300 (7)	
Br3	0.96192 (2)	0.05035 (8)	0.82512 (3)	0.04011 (16)	
C4	0.91689 (15)	0.4331 (7)	0.9832 (3)	0.0387 (8)	
H4	0.9468	0.3866	1.0383	0.046*	
C5	0.88018 (16)	0.6257 (8)	1.0131 (3)	0.0422 (9)	
H5	0.8855	0.7119	1.0884	0.051*	
C6	0.83486 (14)	0.6942 (7)	0.9320 (3)	0.0375 (8)	
H6	0.8101	0.8266	0.9525	0.045*	
C11	0.77814 (13)	0.6452 (7)	0.7322 (3)	0.0307 (7)	
O11	0.76470 (12)	0.8823 (5)	0.7208 (3)	0.0490 (7)	
N11	0.75247 (12)	0.4425 (6)	0.6688 (3)	0.0327 (6)	
H11	0.7634 (15)	0.274 (7)	0.689 (3)	0.039*	
S11	0.55928 (5)	0.8448 (3)	0.49883 (12)	0.0659 (4)	
C12	0.53824 (18)	0.6976 (10)	0.3595 (4)	0.0604 (12)	
H12	0.5048	0.7447	0.3163	0.072*	
N13	0.57051 (15)	0.5206 (8)	0.3178 (3)	0.0594 (10)	
C13A	0.61798 (16)	0.4886 (8)	0.4010 (3)	0.0421 (9)	
C14	0.66075 (17)	0.3153 (10)	0.3870 (3)	0.0530 (11)	
H14	0.6603	0.2073	0.3177	0.064*	
C15	0.70478 (16)	0.3012 (8)	0.4767 (3)	0.0455 (9)	
H15	0.7336	0.1800	0.4687	0.055*	
C16	0.70600 (14)	0.4690 (7)	0.5795 (3)	0.0310 (7)	
C17	0.66363 (14)	0.6445 (7)	0.5954 (3)	0.0375 (8)	
H17	0.6645	0.7552	0.6639	0.045*	
C17A	0.61882 (14)	0.6514 (8)	0.5046 (3)	0.0397 (8)	

### N-(Benzo[d]thiazol-6-yl)-3-bromobenzamide (I) Atomic displacement parameters (Å^2^)

**Table:**

	*U*^11^	*U*^22^	*U*^33^	*U*^12^	*U*^13^	*U*^23^
C1	0.0266 (16)	0.0218 (16)	0.0363 (16)	0.0008 (13)	−0.0016 (12)	0.0046 (14)
C2	0.0307 (17)	0.0245 (16)	0.0313 (16)	−0.0011 (13)	−0.0026 (12)	0.0009 (13)
C3	0.0270 (16)	0.0284 (17)	0.0338 (16)	0.0008 (14)	−0.0008 (12)	0.0076 (14)
Br3	0.0313 (2)	0.0432 (3)	0.0450 (2)	0.01066 (16)	−0.00006 (14)	0.00559 (16)
C4	0.040 (2)	0.045 (2)	0.0296 (16)	−0.0045 (17)	−0.0045 (14)	0.0042 (16)
C5	0.047 (2)	0.046 (2)	0.0321 (18)	0.0028 (18)	−0.0032 (15)	−0.0062 (16)
C6	0.038 (2)	0.035 (2)	0.0389 (18)	0.0046 (16)	0.0054 (14)	−0.0022 (16)
C11	0.0263 (17)	0.0246 (16)	0.0404 (18)	0.0033 (14)	−0.0011 (13)	0.0048 (14)
O11	0.0486 (17)	0.0207 (12)	0.0733 (19)	0.0058 (11)	−0.0175 (13)	0.0014 (12)
N11	0.0316 (16)	0.0199 (14)	0.0444 (16)	0.0061 (12)	−0.0078 (12)	0.0036 (13)
S11	0.0404 (6)	0.0748 (8)	0.0777 (8)	0.0279 (6)	−0.0188 (5)	−0.0133 (7)
C12	0.038 (2)	0.077 (3)	0.062 (3)	0.013 (2)	−0.0154 (18)	0.006 (2)
N13	0.039 (2)	0.087 (3)	0.049 (2)	0.0027 (19)	−0.0172 (16)	0.0004 (19)
C13A	0.036 (2)	0.054 (2)	0.0348 (18)	0.0020 (17)	−0.0072 (15)	0.0039 (16)
C14	0.045 (2)	0.069 (3)	0.043 (2)	0.008 (2)	−0.0041 (16)	−0.016 (2)
C15	0.036 (2)	0.047 (2)	0.052 (2)	0.0107 (18)	−0.0015 (16)	−0.0086 (19)
C16	0.0284 (17)	0.0274 (17)	0.0358 (17)	0.0020 (14)	−0.0047 (13)	0.0043 (14)
C17	0.0339 (19)	0.0341 (18)	0.0426 (19)	0.0060 (16)	−0.0063 (14)	−0.0040 (16)
C17A	0.0290 (18)	0.041 (2)	0.047 (2)	0.0100 (16)	−0.0061 (14)	0.0043 (17)

### N-(Benzo[d]thiazol-6-yl)-3-bromobenzamide (I) Geometric parameters (Å, º)

**Table:**

C1—C6	1.387 (4)	N11—H11	0.90 (4)
C1—C2	1.388 (5)	S11—C17A	1.727 (4)
C1—C11	1.510 (4)	S11—C12	1.730 (5)
C2—C3	1.385 (4)	C12—N13	1.287 (6)
C2—H2	0.9300	C12—H12	0.9300
C3—C4	1.373 (5)	N13—C13A	1.410 (5)
C3—Br3	1.903 (3)	C13A—C14	1.365 (6)
C4—C5	1.364 (5)	C13A—C17A	1.394 (5)
C4—H4	0.9300	C14—C15	1.385 (5)
C5—C6	1.390 (5)	C14—H14	0.9300
C5—H5	0.9300	C15—C16	1.400 (5)
C6—H6	0.9300	C15—H15	0.9300
C11—O11	1.221 (4)	C16—C17	1.368 (5)
C11—N11	1.341 (4)	C17—C17A	1.405 (4)
N11—C16	1.429 (4)	C17—H17	0.9300
			
C6—C1—C2	120.2 (3)	C17A—S11—C12	88.6 (2)
C6—C1—C11	118.6 (3)	N13—C12—S11	117.6 (3)
C2—C1—C11	121.2 (3)	N13—C12—H12	121.2
C3—C2—C1	118.8 (3)	S11—C12—H12	121.2
C3—C2—H2	120.6	C12—N13—C13A	109.3 (4)
C1—C2—H2	120.6	C14—C13A—C17A	120.1 (3)
C4—C3—C2	121.2 (3)	C14—C13A—N13	125.4 (4)
C4—C3—Br3	120.4 (2)	C17A—C13A—N13	114.5 (4)
C2—C3—Br3	118.4 (2)	C13A—C14—C15	119.6 (4)
C5—C4—C3	119.8 (3)	C13A—C14—H14	120.2
C5—C4—H4	120.1	C15—C14—H14	120.2
C3—C4—H4	120.1	C14—C15—C16	120.1 (3)
C4—C5—C6	120.6 (3)	C14—C15—H15	120.0
C4—C5—H5	119.7	C16—C15—H15	120.0
C6—C5—H5	119.7	C17—C16—C15	121.4 (3)
C1—C6—C5	119.4 (3)	C17—C16—N11	121.5 (3)
C1—C6—H6	120.3	C15—C16—N11	117.1 (3)
C5—C6—H6	120.3	C16—C17—C17A	117.6 (3)
O11—C11—N11	124.0 (3)	C16—C17—H17	121.2
O11—C11—C1	120.6 (3)	C17A—C17—H17	121.2
N11—C11—C1	115.4 (3)	C13A—C17A—C17	121.2 (3)
C11—N11—C16	125.9 (3)	C13A—C17A—S11	110.0 (3)
C11—N11—H11	117 (2)	C17—C17A—S11	128.8 (3)
C16—N11—H11	117 (2)		
			
C6—C1—C2—C3	−0.8 (5)	C12—N13—C13A—C17A	−0.3 (6)
C11—C1—C2—C3	177.2 (3)	C17A—C13A—C14—C15	0.4 (7)
C1—C2—C3—C4	2.2 (5)	N13—C13A—C14—C15	−179.2 (4)
C1—C2—C3—Br3	−177.3 (2)	C13A—C14—C15—C16	−1.7 (7)
C2—C3—C4—C5	−2.2 (5)	C14—C15—C16—C17	1.6 (6)
Br3—C3—C4—C5	177.3 (3)	C14—C15—C16—N11	179.6 (4)
C3—C4—C5—C6	0.8 (6)	C11—N11—C16—C17	−39.9 (5)
C2—C1—C6—C5	−0.5 (5)	C11—N11—C16—C15	142.2 (4)
C11—C1—C6—C5	−178.6 (3)	C15—C16—C17—C17A	−0.3 (6)
C4—C5—C6—C1	0.5 (6)	N11—C16—C17—C17A	−178.1 (3)
C6—C1—C11—O11	39.0 (5)	C14—C13A—C17A—C17	1.0 (6)
C2—C1—C11—O11	−139.0 (4)	N13—C13A—C17A—C17	−179.4 (4)
C6—C1—C11—N11	−141.9 (3)	C14—C13A—C17A—S11	−179.1 (3)
C2—C1—C11—N11	40.0 (4)	N13—C13A—C17A—S11	0.6 (5)
O11—C11—N11—C16	−0.2 (6)	C16—C17—C17A—C13A	−1.0 (6)
C1—C11—N11—C16	−179.2 (3)	C16—C17—C17A—S11	179.0 (3)
C17A—S11—C12—N13	0.3 (4)	C12—S11—C17A—C13A	−0.5 (3)
S11—C12—N13—C13A	0.0 (6)	C12—S11—C17A—C17	179.5 (4)
C12—N13—C13A—C14	179.3 (4)		

### N-(Benzo[d]thiazol-6-yl)-3-bromobenzamide (I) Hydrogen-bond geometry (Å, º)

**Table:**

*D*—H···*A*	*D*—H	H···*A*	*D*···*A*	*D*—H···*A*
N11—H11···O11^i^	0.90 (4)	1.97 (3)	2.840 (4)	164 (3)
C12—H12···N13^ii^	0.93	2.62	3.512 (6)	161

Symmetry codes: (i) *x*, *y*−1, *z*; (ii) −*x*+1, *y*+1/2, −*z*+1/2.

### N-(6-Methoxybenzo[d]thiazol-2-yl)-2-nitrobenzamide (II) Crystal data

**Table:**

C_15_H_11_N_3_O_4_S	*D*_x_ = 1.475 Mg m^−^^3^
*M**_r_* = 329.33	Mo *K*α radiation, λ = 0.71073 Å
Orthorhombic, *P**n**a*2_1_	Cell parameters from 4785 reflections
*a* = 20.085 (2) Å	θ = 2.9–28.8°
*b* = 20.165 (2) Å	µ = 0.24 mm^−^^1^
*c* = 7.3220 (6) Å	*T* = 296 K
*V* = 2965.5 (5) Å^3^	Needle, yellow
*Z* = 8	0.50 × 0.12 × 0.10 mm
*F*(000) = 1360	

### N-(6-Methoxybenzo[d]thiazol-2-yl)-2-nitrobenzamide (II) Data collection

**Table:**

Oxford Diffraction Xcalibur CCD diffractometer	4784 independent reflections
Radiation source: Enhance (Mo) X-ray Source	2969 reflections with *I* > 2σ(*I*)
Graphite monochromator	*R*_int_ = 0.040
ω scans	θ_max_ = 28.0°, θ_min_ = 2.9°
Absorption correction: multi-scan (CrysalisRed; Oxford Diffraction, 2009)	*h* = −22→25
*T*_min_ = 0.787, *T*_max_ = 0.976	*k* = −25→23
8012 measured reflections	*l* = −3→9

### N-(6-Methoxybenzo[d]thiazol-2-yl)-2-nitrobenzamide (II) Refinement

**Table:**

Refinement on *F*^2^	Hydrogen site location: mixed
Least-squares matrix: full	H atoms treated by a mixture of independent and constrained refinement
*R*[*F*^2^ > 2σ(*F*^2^)] = 0.049	*w* = 1/[σ^2^(*F*_o_^2^) + (0.019*P*)^2^ + 0.5603*P*] where *P* = (*F*_o_^2^ + 2*F*_c_^2^)/3
*wR*(*F*^2^) = 0.075	(Δ/σ)_max_ < 0.001
*S* = 1.05	Δρ_max_ = 0.19 e Å^−^^3^
4784 reflections	Δρ_min_ = −0.22 e Å^−^^3^
423 parameters	Absolute structure: Flack x parameter (Parsons *et al.*, 2013)
1 restraint	Absolute structure parameter: 0.02 (5)
Primary atom site location: difference Fourier map	

### N-(6-Methoxybenzo[d]thiazol-2-yl)-2-nitrobenzamide (II) Special details

**Table:**

Geometry. All esds (except the esd in the dihedral angle between two l.s. planes) are estimated using the full covariance matrix. The cell esds are taken into account individually in the estimation of esds in distances, angles and torsion angles; correlations between esds in cell parameters are only used when they are defined by crystal symmetry. An approximate (isotropic) treatment of cell esds is used for estimating esds involving l.s. planes.

### N-(6-Methoxybenzo[d]thiazol-2-yl)-2-nitrobenzamide (II) Fractional atomic coordinates and isotropic or equivalent isotropic displacement parameters (Å^2^)

**Table:**

	*x*	*y*	*z*	*U*_iso_*/*U*_eq_	
C11	0.6647 (2)	0.4210 (2)	0.1128 (7)	0.0416 (11)	
C12	0.7079 (3)	0.4721 (2)	0.0750 (7)	0.0485 (13)	
C13	0.7447 (3)	0.4762 (3)	−0.0845 (8)	0.0585 (15)	
H13	0.7729	0.5119	−0.1054	0.070*	
C14	0.7386 (3)	0.4264 (3)	−0.2113 (8)	0.0633 (15)	
H14	0.7617	0.4286	−0.3212	0.076*	
C15	0.6983 (3)	0.3734 (3)	−0.1744 (7)	0.0578 (15)	
H15	0.6958	0.3387	−0.2576	0.069*	
C16	0.6612 (3)	0.3706 (2)	−0.0159 (7)	0.0494 (13)	
H16	0.6334	0.3344	0.0050	0.059*	
C111	0.6168 (3)	0.4224 (3)	0.2709 (7)	0.0491 (13)	
O111	0.5820 (2)	0.47044 (17)	0.3001 (5)	0.0719 (11)	
N111	0.6129 (2)	0.36582 (19)	0.3718 (6)	0.0468 (12)	
H111	0.642 (2)	0.339 (2)	0.348 (7)	0.056*	
N121	0.7207 (2)	0.5232 (2)	0.2121 (8)	0.0714 (15)	
O121	0.7199 (3)	0.5076 (2)	0.3713 (7)	0.1044 (17)	
O122	0.7336 (2)	0.57908 (19)	0.1580 (7)	0.1033 (16)	
S111	0.52655 (6)	0.42259 (6)	0.61418 (19)	0.0495 (3)	
C112	0.5731 (2)	0.3570 (2)	0.5239 (6)	0.0394 (12)	
N113	0.57191 (18)	0.30181 (16)	0.6124 (6)	0.0433 (9)	
C13A	0.5335 (2)	0.3086 (2)	0.7691 (7)	0.0410 (12)	
C114	0.5238 (2)	0.2610 (2)	0.9031 (7)	0.0498 (13)	
H114	0.5421	0.2189	0.8892	0.060*	
C115	0.4873 (2)	0.2755 (3)	1.0550 (7)	0.0549 (15)	
H115	0.4815	0.2434	1.1447	0.066*	
C116	0.4585 (2)	0.3382 (2)	1.0778 (7)	0.0512 (13)	
C117	0.4667 (2)	0.3862 (2)	0.9472 (7)	0.0459 (13)	
H117	0.4474	0.4278	0.9603	0.055*	
C17A	0.5048 (2)	0.3705 (2)	0.7939 (6)	0.0421 (12)	
O116	0.42262 (18)	0.34579 (18)	1.2344 (5)	0.0687 (11)	
C118	0.3883 (3)	0.4068 (3)	1.2602 (7)	0.0763 (18)	
H18A	0.3605	0.4037	1.3665	0.115*	
H18B	0.3613	0.4159	1.1551	0.115*	
H18C	0.4200	0.4419	1.2765	0.115*	
C21	0.5587 (2)	0.10314 (18)	0.6179 (7)	0.0356 (10)	
C22	0.4907 (2)	0.09346 (19)	0.6067 (7)	0.0391 (11)	
C23	0.4527 (3)	0.0724 (2)	0.7525 (7)	0.0475 (13)	
H23	0.4071	0.0658	0.7400	0.057*	
C24	0.4839 (3)	0.0612 (2)	0.9165 (7)	0.0506 (14)	
H24	0.4592	0.0472	1.0168	0.061*	
C25	0.5511 (3)	0.0706 (2)	0.9332 (7)	0.0524 (14)	
H25	0.5717	0.0631	1.0451	0.063*	
C26	0.5889 (3)	0.0912 (2)	0.7851 (6)	0.0449 (13)	
H26	0.6346	0.0971	0.7979	0.054*	
C211	0.6042 (2)	0.1170 (2)	0.4589 (6)	0.0404 (12)	
O211	0.61896 (16)	0.07405 (15)	0.3509 (4)	0.0478 (9)	
N211	0.6313 (2)	0.17844 (19)	0.4542 (5)	0.0428 (10)	
H211	0.617 (2)	0.210 (2)	0.522 (5)	0.051*	
N221	0.4558 (2)	0.1058 (2)	0.4325 (6)	0.0519 (11)	
O221	0.48494 (19)	0.13925 (18)	0.3175 (5)	0.0705 (12)	
O222	0.4004 (2)	0.0816 (2)	0.4107 (6)	0.0818 (13)	
S211	0.70172 (6)	0.15015 (5)	0.14183 (17)	0.0474 (3)	
C212	0.6797 (2)	0.1980 (2)	0.3302 (6)	0.0372 (12)	
N213	0.71002 (18)	0.25434 (17)	0.3486 (5)	0.0384 (9)	
C23A	0.7553 (2)	0.2629 (2)	0.2067 (6)	0.0375 (12)	
C214	0.7983 (2)	0.3165 (2)	0.1862 (6)	0.0449 (13)	
H214	0.8000	0.3498	0.2739	0.054*	
C215	0.8381 (2)	0.3192 (2)	0.0346 (7)	0.0488 (14)	
H215	0.8683	0.3539	0.0229	0.059*	
C216	0.8344 (2)	0.2713 (2)	−0.1031 (7)	0.0468 (13)	
C217	0.7948 (2)	0.2163 (2)	−0.0808 (6)	0.0463 (13)	
H217	0.7942	0.1825	−0.1671	0.056*	
C27A	0.7560 (2)	0.2131 (2)	0.0749 (6)	0.0393 (12)	
O216	0.87332 (17)	0.28389 (17)	−0.2525 (5)	0.0614 (10)	
C218	0.8728 (3)	0.2358 (3)	−0.3963 (7)	0.0652 (15)	
H28A	0.9029	0.2494	−0.4911	0.098*	
H28B	0.8286	0.2321	−0.4453	0.098*	
H28C	0.8866	0.1935	−0.3489	0.098*	

### N-(6-Methoxybenzo[d]thiazol-2-yl)-2-nitrobenzamide (II) Atomic displacement parameters (Å^2^)

**Table:**

	*U*^11^	*U*^22^	*U*^33^	*U*^12^	*U*^13^	*U*^23^
C11	0.047 (3)	0.038 (2)	0.040 (3)	0.000 (2)	−0.002 (3)	0.007 (3)
C12	0.058 (3)	0.039 (3)	0.049 (3)	0.000 (3)	−0.004 (3)	0.003 (2)
C13	0.055 (4)	0.055 (4)	0.066 (4)	−0.004 (3)	0.005 (3)	0.013 (3)
C14	0.061 (4)	0.082 (4)	0.048 (3)	0.001 (4)	0.002 (3)	0.010 (3)
C15	0.058 (4)	0.069 (4)	0.047 (3)	0.002 (3)	−0.008 (3)	−0.009 (3)
C16	0.046 (3)	0.050 (3)	0.053 (3)	0.001 (3)	−0.011 (3)	−0.002 (3)
C111	0.054 (4)	0.044 (3)	0.049 (3)	−0.003 (3)	0.001 (3)	0.002 (3)
O111	0.097 (3)	0.048 (2)	0.071 (3)	0.026 (2)	0.021 (2)	0.0119 (19)
N111	0.052 (3)	0.035 (3)	0.054 (3)	0.006 (2)	0.007 (2)	0.000 (2)
N121	0.083 (4)	0.050 (3)	0.081 (4)	−0.005 (3)	0.001 (3)	−0.006 (3)
O121	0.159 (5)	0.088 (4)	0.066 (3)	−0.017 (3)	−0.020 (3)	−0.016 (3)
O122	0.136 (4)	0.047 (2)	0.127 (4)	−0.024 (3)	0.019 (3)	−0.014 (3)
S111	0.0575 (8)	0.0389 (6)	0.0519 (8)	0.0095 (6)	0.0048 (8)	0.0053 (7)
C112	0.036 (3)	0.037 (3)	0.045 (3)	−0.001 (2)	0.001 (2)	−0.004 (2)
N113	0.048 (2)	0.030 (2)	0.052 (2)	−0.0026 (18)	0.003 (2)	0.004 (2)
C13A	0.039 (3)	0.034 (3)	0.050 (3)	−0.004 (2)	0.000 (3)	0.000 (2)
C114	0.055 (4)	0.032 (3)	0.062 (4)	0.001 (3)	0.003 (3)	0.003 (3)
C115	0.059 (4)	0.045 (3)	0.060 (4)	−0.007 (3)	0.008 (3)	0.013 (2)
C116	0.053 (3)	0.043 (3)	0.057 (4)	−0.003 (3)	−0.001 (3)	−0.003 (3)
C117	0.051 (3)	0.036 (3)	0.051 (3)	0.002 (3)	0.003 (3)	0.003 (2)
C17A	0.043 (3)	0.033 (3)	0.050 (3)	−0.003 (2)	−0.005 (3)	0.003 (2)
O116	0.087 (3)	0.060 (3)	0.060 (2)	0.007 (2)	0.028 (2)	0.008 (2)
C118	0.087 (5)	0.071 (4)	0.071 (4)	0.014 (4)	0.027 (4)	−0.002 (3)
C21	0.037 (3)	0.030 (2)	0.040 (3)	−0.007 (2)	0.002 (3)	−0.002 (2)
C22	0.048 (3)	0.034 (3)	0.035 (3)	0.006 (2)	0.002 (3)	0.005 (2)
C23	0.041 (3)	0.045 (3)	0.056 (4)	0.004 (3)	0.006 (3)	−0.001 (3)
C24	0.062 (4)	0.045 (3)	0.045 (3)	0.000 (3)	0.013 (3)	0.006 (2)
C25	0.071 (4)	0.046 (3)	0.040 (3)	−0.004 (3)	−0.009 (3)	0.001 (3)
C26	0.043 (3)	0.050 (3)	0.042 (3)	−0.005 (3)	−0.008 (3)	−0.003 (3)
C211	0.043 (3)	0.038 (3)	0.040 (3)	−0.002 (3)	−0.006 (3)	0.002 (2)
O211	0.060 (2)	0.0379 (18)	0.045 (2)	−0.0039 (17)	0.0096 (18)	−0.0089 (15)
N211	0.051 (3)	0.035 (2)	0.043 (3)	−0.004 (2)	0.009 (2)	−0.0040 (19)
N221	0.052 (3)	0.047 (3)	0.057 (3)	0.010 (2)	−0.012 (3)	0.001 (2)
O221	0.077 (3)	0.080 (3)	0.054 (2)	0.005 (2)	−0.007 (2)	0.027 (2)
O222	0.058 (3)	0.092 (3)	0.096 (3)	−0.005 (3)	−0.035 (2)	0.017 (2)
S211	0.0530 (8)	0.0404 (7)	0.0487 (8)	−0.0104 (6)	0.0091 (7)	−0.0073 (7)
C212	0.041 (3)	0.032 (3)	0.039 (3)	−0.002 (2)	0.002 (2)	0.002 (2)
N213	0.037 (2)	0.034 (2)	0.044 (2)	−0.0061 (19)	0.008 (2)	−0.0021 (18)
C23A	0.035 (3)	0.032 (3)	0.045 (3)	−0.002 (2)	−0.006 (2)	−0.003 (2)
C214	0.041 (3)	0.045 (3)	0.049 (3)	−0.003 (3)	0.004 (3)	−0.005 (2)
C215	0.043 (3)	0.048 (3)	0.055 (4)	−0.014 (3)	−0.004 (3)	0.005 (3)
C216	0.039 (3)	0.056 (3)	0.046 (3)	−0.003 (3)	0.007 (3)	0.007 (3)
C217	0.051 (3)	0.043 (3)	0.045 (3)	−0.003 (3)	0.003 (3)	−0.005 (2)
C27A	0.034 (3)	0.037 (3)	0.047 (3)	−0.006 (2)	0.004 (2)	0.002 (2)
O216	0.063 (3)	0.068 (3)	0.053 (2)	−0.017 (2)	0.012 (2)	0.0009 (19)
C218	0.058 (4)	0.088 (4)	0.050 (3)	−0.010 (3)	0.011 (3)	0.002 (3)

### N-(6-Methoxybenzo[d]thiazol-2-yl)-2-nitrobenzamide (II) Geometric parameters (Å, º)

**Table:**

C11—C12	1.374 (6)	C21—C22	1.382 (5)
C11—C16	1.389 (6)	C21—C26	1.388 (6)
C11—C111	1.507 (7)	C21—C211	1.507 (6)
C12—C13	1.384 (6)	C22—C23	1.379 (6)
C12—N121	1.461 (6)	C22—N221	1.477 (6)
C13—C14	1.373 (7)	C23—C24	1.373 (6)
C13—H13	0.9300	C23—H23	0.9300
C14—C15	1.367 (7)	C24—C25	1.370 (6)
C14—H14	0.9300	C24—H24	0.9300
C15—C16	1.381 (7)	C25—C26	1.387 (6)
C15—H15	0.9300	C25—H25	0.9300
C16—H16	0.9300	C26—H26	0.9300
C111—O111	1.214 (5)	C211—O211	1.209 (5)
C111—N111	1.361 (6)	C211—N211	1.354 (5)
N111—C112	1.383 (6)	N211—C212	1.388 (6)
N111—H111	0.81 (4)	N211—H211	0.86 (4)
N121—O121	1.207 (6)	N221—O222	1.225 (5)
N121—O122	1.223 (5)	N221—O221	1.228 (5)
S111—C17A	1.739 (5)	S211—C212	1.740 (4)
S111—C112	1.749 (5)	S211—C27A	1.744 (4)
C112—N113	1.288 (5)	C212—N213	1.296 (5)
N113—C13A	1.389 (6)	N213—C23A	1.391 (5)
C13A—C17A	1.387 (5)	C23A—C27A	1.392 (6)
C13A—C114	1.387 (6)	C23A—C214	1.393 (6)
C114—C115	1.364 (6)	C214—C215	1.368 (6)
C114—H114	0.9300	C214—H214	0.9300
C115—C116	1.400 (6)	C215—C216	1.399 (6)
C115—H115	0.9300	C215—H215	0.9300
C116—O116	1.364 (6)	C216—O216	1.368 (5)
C116—C117	1.370 (6)	C216—C217	1.374 (6)
C117—C17A	1.395 (6)	C217—C27A	1.383 (6)
C117—H117	0.9300	C217—H217	0.9300
O116—C118	1.422 (5)	O216—C218	1.432 (6)
C118—H18A	0.9600	C218—H28A	0.9600
C118—H18B	0.9600	C218—H28B	0.9600
C118—H18C	0.9600	C218—H28C	0.9600
			
C12—C11—C16	116.4 (5)	C22—C21—C26	117.4 (4)
C12—C11—C111	123.0 (5)	C22—C21—C211	125.5 (4)
C16—C11—C111	120.1 (4)	C26—C21—C211	116.6 (4)
C11—C12—C13	123.4 (5)	C23—C22—C21	123.0 (5)
C11—C12—N121	120.1 (5)	C23—C22—N221	117.3 (4)
C13—C12—N121	116.3 (5)	C21—C22—N221	119.7 (4)
C14—C13—C12	118.7 (5)	C24—C23—C22	118.4 (5)
C14—C13—H13	120.7	C24—C23—H23	120.8
C12—C13—H13	120.7	C22—C23—H23	120.8
C15—C14—C13	119.3 (5)	C25—C24—C23	120.3 (5)
C15—C14—H14	120.3	C25—C24—H24	119.8
C13—C14—H14	120.3	C23—C24—H24	119.8
C14—C15—C16	121.3 (5)	C24—C25—C26	120.8 (5)
C14—C15—H15	119.4	C24—C25—H25	119.6
C16—C15—H15	119.4	C26—C25—H25	119.6
C15—C16—C11	120.8 (5)	C25—C26—C21	120.1 (5)
C15—C16—H16	119.6	C25—C26—H26	119.9
C11—C16—H16	119.6	C21—C26—H26	119.9
O111—C111—N111	122.7 (5)	O211—C211—N211	122.7 (5)
O111—C111—C11	121.2 (5)	O211—C211—C21	121.4 (4)
N111—C111—C11	116.0 (5)	N211—C211—C21	115.6 (4)
C111—N111—C112	125.3 (4)	C211—N211—C212	123.9 (4)
C111—N111—H111	113 (4)	C211—N211—H211	122 (3)
C112—N111—H111	121 (4)	C212—N211—H211	114 (3)
O121—N121—O122	123.8 (5)	O222—N221—O221	124.2 (5)
O121—N121—C12	118.5 (5)	O222—N221—C22	118.5 (5)
O122—N121—C12	117.7 (5)	O221—N221—C22	117.3 (4)
C17A—S111—C112	87.9 (2)	C212—S211—C27A	88.7 (2)
N113—C112—N111	121.8 (4)	N213—C212—N211	120.7 (4)
N113—C112—S111	117.0 (4)	N213—C212—S211	116.7 (3)
N111—C112—S111	121.1 (4)	N211—C212—S211	122.6 (3)
C112—N113—C13A	109.9 (4)	C212—N213—C23A	109.7 (4)
C17A—C13A—C114	118.2 (4)	N213—C23A—C27A	115.8 (4)
C17A—C13A—N113	115.3 (4)	N213—C23A—C214	125.6 (4)
C114—C13A—N113	126.4 (4)	C27A—C23A—C214	118.6 (4)
C115—C114—C13A	120.2 (5)	C215—C214—C23A	118.8 (4)
C115—C114—H114	119.9	C215—C214—H214	120.6
C13A—C114—H114	119.9	C23A—C214—H214	120.6
C114—C115—C116	120.9 (5)	C214—C215—C216	121.8 (5)
C114—C115—H115	119.6	C214—C215—H215	119.1
C116—C115—H115	119.6	C216—C215—H215	119.1
O116—C116—C117	124.8 (5)	O216—C216—C217	125.1 (4)
O116—C116—C115	114.8 (5)	O216—C216—C215	114.7 (4)
C117—C116—C115	120.3 (5)	C217—C216—C215	120.1 (4)
C116—C117—C17A	117.9 (4)	C216—C217—C27A	117.5 (4)
C116—C117—H117	121.1	C216—C217—H217	121.2
C17A—C117—H117	121.1	C27A—C217—H217	121.2
C13A—C17A—C117	122.5 (4)	C217—C27A—C23A	122.9 (4)
C13A—C17A—S111	109.9 (4)	C217—C27A—S211	128.2 (4)
C117—C17A—S111	127.6 (4)	C23A—C27A—S211	108.9 (3)
C116—O116—C118	117.7 (4)	C216—O216—C218	117.3 (4)
O116—C118—H18A	109.5	O216—C218—H28A	109.5
O116—C118—H18B	109.5	O216—C218—H28B	109.5
H18A—C118—H18B	109.5	H28A—C218—H28B	109.5
O116—C118—H18C	109.5	O216—C218—H28C	109.5
H18A—C118—H18C	109.5	H28A—C218—H28C	109.5
H18B—C118—H18C	109.5	H28B—C218—H28C	109.5
			
C16—C11—C12—C13	2.6 (7)	C26—C21—C22—C23	−0.5 (6)
C111—C11—C12—C13	−169.6 (5)	C211—C21—C22—C23	171.1 (4)
C16—C11—C12—N121	−172.8 (4)	C26—C21—C22—N221	179.6 (4)
C111—C11—C12—N121	15.0 (7)	C211—C21—C22—N221	−8.8 (6)
C11—C12—C13—C14	−0.9 (8)	C21—C22—C23—C24	0.7 (7)
N121—C12—C13—C14	174.7 (5)	N221—C22—C23—C24	−179.3 (4)
C12—C13—C14—C15	−2.0 (8)	C22—C23—C24—C25	−0.3 (7)
C13—C14—C15—C16	3.1 (8)	C23—C24—C25—C26	−0.3 (7)
C14—C15—C16—C11	−1.3 (8)	C24—C25—C26—C21	0.6 (7)
C12—C11—C16—C15	−1.5 (7)	C22—C21—C26—C25	−0.2 (6)
C111—C11—C16—C15	170.9 (4)	C211—C21—C26—C25	−172.6 (4)
C12—C11—C111—O111	47.8 (7)	C22—C21—C211—O211	−73.2 (6)
C16—C11—C111—O111	−124.1 (5)	C26—C21—C211—O211	98.5 (5)
C12—C11—C111—N111	−135.1 (5)	C22—C21—C211—N211	111.9 (5)
C16—C11—C111—N111	53.0 (7)	C26—C21—C211—N211	−76.5 (5)
O111—C111—N111—C112	−4.1 (8)	O211—C211—N211—C212	−2.0 (7)
C11—C111—N111—C112	178.8 (4)	C21—C211—N211—C212	172.8 (4)
C11—C12—N121—O121	33.1 (8)	C23—C22—N221—O222	−18.2 (6)
C13—C12—N121—O121	−142.7 (6)	C21—C22—N221—O222	161.7 (4)
C11—C12—N121—O122	−148.9 (5)	C23—C22—N221—O221	162.7 (4)
C13—C12—N121—O122	35.4 (7)	C21—C22—N221—O221	−17.4 (6)
C111—N111—C112—N113	179.8 (5)	C211—N211—C212—N213	−170.5 (4)
C111—N111—C112—S111	−5.0 (7)	C211—N211—C212—S211	10.9 (6)
C17A—S111—C112—N113	1.6 (4)	C27A—S211—C212—N213	−1.8 (4)
C17A—S111—C112—N111	−173.9 (4)	C27A—S211—C212—N211	176.9 (4)
N111—C112—N113—C13A	173.7 (4)	N211—C212—N213—C23A	−179.2 (4)
S111—C112—N113—C13A	−1.8 (5)	S211—C212—N213—C23A	−0.4 (5)
C112—N113—C13A—C17A	1.1 (6)	C212—N213—C23A—C27A	3.3 (5)
C112—N113—C13A—C114	−176.2 (5)	C212—N213—C23A—C214	−177.4 (4)
C17A—C13A—C114—C115	−0.5 (7)	N213—C23A—C214—C215	−177.2 (4)
N113—C13A—C114—C115	176.7 (4)	C27A—C23A—C214—C215	2.0 (6)
C13A—C114—C115—C116	0.7 (8)	C23A—C214—C215—C216	2.8 (7)
C114—C115—C116—O116	179.1 (4)	C214—C215—C216—O216	174.8 (4)
C114—C115—C116—C117	−0.1 (8)	C214—C215—C216—C217	−6.3 (7)
O116—C116—C117—C17A	−179.8 (4)	O216—C216—C217—C27A	−176.7 (4)
C115—C116—C117—C17A	−0.7 (7)	C215—C216—C217—C27A	4.5 (7)
C114—C13A—C17A—C117	−0.3 (7)	C216—C217—C27A—C23A	0.4 (7)
N113—C13A—C17A—C117	−177.9 (4)	C216—C217—C27A—S211	−179.4 (4)
C114—C13A—C17A—S111	177.6 (4)	N213—C23A—C27A—C217	175.6 (4)
N113—C13A—C17A—S111	0.1 (5)	C214—C23A—C27A—C217	−3.7 (7)
C116—C117—C17A—C13A	0.9 (7)	N213—C23A—C27A—S211	−4.5 (5)
C116—C117—C17A—S111	−176.6 (4)	C214—C23A—C27A—S211	176.2 (3)
C112—S111—C17A—C13A	−0.8 (3)	C212—S211—C27A—C217	−176.8 (4)
C112—S111—C17A—C117	177.0 (5)	C212—S211—C27A—C23A	3.4 (4)
C117—C116—O116—C118	3.3 (7)	C217—C216—O216—C218	−0.3 (7)
C115—C116—O116—C118	−175.8 (4)	C215—C216—O216—C218	178.6 (4)

### N-(6-Methoxybenzo[d]thiazol-2-yl)-2-nitrobenzamide (II) Hydrogen-bond geometry (Å, º)

**Table:**

*D*—H···*A*	*D*—H	H···*A*	*D*···*A*	*D*—H···*A*
N111—H111···N213	0.82 (4)	2.19 (4)	2.981 (5)	165 (4)
N211—H211···N113	0.86 (4)	2.17 (4)	2.992 (5)	162 (4)
C13—H13···O211^i^	0.93	2.53	3.408 (7)	158
C25—H25···O211^ii^	0.93	2.44	3.349 (6)	165
C115—H115···O221^ii^	0.93	2.45	3.353 (7)	163
C117—H117···O111^iii^	0.93	2.44	3.236 (5)	144
C217—H217···O122^iv^	0.93	2.51	3.412 (6)	164
C16—H16···*Cg*1^v^	0.93	2.84	3.484 (6)	128

Symmetry codes: (i) −*x*+3/2, *y*+1/2, *z*−1/2; (ii) *x*, *y*, *z*+1; (iii) −*x*+1, −*y*+1, *z*+1/2; (iv) −*x*+3/2, *y*−1/2, *z*−1/2; (v) *x*, *y*, *z*−1.

### 5-Cyclopropyl-N-(6-methoxybenzo[d]thiazol-2-yl)isoxazole-3-carboxamide (III) Crystal data

**Table:**

C_15_H_13_N_3_O_3_S	*F*(000) = 1312
*M**_r_* = 315.34	*D*_x_ = 1.455 Mg m^−^^3^
Monoclinic, *C*2/*c*	Mo *K*α radiation, λ = 0.71073 Å
*a* = 18.720 (1) Å	Cell parameters from 3118 reflections
*b* = 11.5255 (8) Å	θ = 2.9–27.8°
*c* = 14.7905 (9) Å	µ = 0.24 mm^−^^1^
β = 115.52 (1)°	*T* = 296 K
*V* = 2879.8 (4) Å^3^	Block, colourless
*Z* = 8	0.30 × 0.20 × 0.10 mm

### 5-Cyclopropyl-N-(6-methoxybenzo[d]thiazol-2-yl)isoxazole-3-carboxamide (III) Data collection

**Table:**

Oxford Diffraction Xcalibur CCD diffractometer	3118 independent reflections
Radiation source: Enhance (Mo) X-ray Source	1730 reflections with *I* > 2σ(*I*)
Graphite monochromator	*R*_int_ = 0.038
ω scans	θ_max_ = 27.8°, θ_min_ = 2.9°
Absorption correction: multi-scan (CrysalisRed; Oxford Diffraction, 2009)	*h* = −24→19
*T*_min_ = 0.908, *T*_max_ = 0.976	*k* = −15→9
5937 measured reflections	*l* = −18→19

### 5-Cyclopropyl-N-(6-methoxybenzo[d]thiazol-2-yl)isoxazole-3-carboxamide (III) Refinement

**Table:**

Refinement on *F*^2^	Primary atom site location: difference Fourier map
Least-squares matrix: full	Hydrogen site location: mixed
*R*[*F*^2^ > 2σ(*F*^2^)] = 0.056	H atoms treated by a mixture of independent and constrained refinement
*wR*(*F*^2^) = 0.119	*w* = 1/[σ^2^(*F*_o_^2^) + (0.0504*P*)^2^] where *P* = (*F*_o_^2^ + 2*F*_c_^2^)/3
*S* = 1.02	(Δ/σ)_max_ = 0.001
3118 reflections	Δρ_max_ = 0.23 e Å^−^^3^
268 parameters	Δρ_min_ = −0.24 e Å^−^^3^
26 restraints	

### 5-Cyclopropyl-N-(6-methoxybenzo[d]thiazol-2-yl)isoxazole-3-carboxamide (III) Special details

**Table:**

Geometry. All esds (except the esd in the dihedral angle between two l.s. planes) are estimated using the full covariance matrix. The cell esds are taken into account individually in the estimation of esds in distances, angles and torsion angles; correlations between esds in cell parameters are only used when they are defined by crystal symmetry. An approximate (isotropic) treatment of cell esds is used for estimating esds involving l.s. planes.

### 5-Cyclopropyl-N-(6-methoxybenzo[d]thiazol-2-yl)isoxazole-3-carboxamide (III) Fractional atomic coordinates and isotropic or equivalent isotropic displacement parameters (Å^2^)

**Table:**

	*x*	*y*	*z*	*U*_iso_*/*U*_eq_	Occ. (<1)
C31B	0.45159 (15)	0.6697 (2)	0.4342 (2)	0.0494 (7)	0.451 (5)
O31B	0.4916 (12)	0.682 (3)	0.5246 (5)	0.056 (3)	0.451 (5)
O1B	0.2557 (4)	0.7800 (10)	0.2806 (7)	0.0511 (13)	0.451 (5)
N2B	0.3373 (6)	0.766 (3)	0.3053 (16)	0.0495 (13)	0.451 (5)
C3B	0.3650 (8)	0.703 (4)	0.387 (3)	0.0441 (12)	0.451 (5)
C4B	0.3059 (8)	0.673 (4)	0.417 (2)	0.0492 (13)	0.451 (5)
H4B	0.3114	0.6272	0.4713	0.059*	0.451 (5)
C5B	0.2392 (4)	0.7242 (13)	0.3499 (8)	0.0446 (17)	0.451 (5)
C51	0.1558 (4)	0.7226 (8)	0.3338 (5)	0.057 (2)	0.451 (5)
H51	0.1464	0.6824	0.3860	0.068*	0.451 (5)
C52	0.0910 (5)	0.7088 (8)	0.2300 (6)	0.055 (3)	0.451 (5)
H52A	0.1058	0.7077	0.1747	0.066*	0.451 (5)
H52B	0.0467	0.6593	0.2214	0.066*	0.451 (5)
C53	0.0996 (4)	0.8175 (6)	0.2835 (5)	0.067 (2)	0.451 (5)
H53A	0.0602	0.8357	0.3077	0.080*	0.451 (5)
H53B	0.1194	0.8841	0.2611	0.080*	0.451 (5)
C31A	0.45159 (15)	0.6697 (2)	0.4342 (2)	0.0494 (7)	0.549 (5)
O31A	0.4786 (9)	0.655 (2)	0.5251 (4)	0.056 (3)	0.549 (5)
O1A	0.2719 (3)	0.8029 (8)	0.2617 (5)	0.0511 (13)	0.549 (5)
N2A	0.3533 (5)	0.778 (2)	0.2975 (13)	0.0495 (13)	0.549 (5)
C3A	0.3683 (6)	0.710 (4)	0.374 (2)	0.0441 (12)	0.549 (5)
C4A	0.3025 (6)	0.690 (3)	0.3936 (17)	0.0492 (13)	0.549 (5)
H4A	0.3002	0.6471	0.4455	0.059*	0.549 (5)
C5A	0.2435 (3)	0.7473 (10)	0.3203 (7)	0.0446 (17)	0.549 (5)
C61	0.1581 (3)	0.7625 (6)	0.2904 (5)	0.058 (2)	0.549 (5)
H61	0.1352	0.8351	0.2554	0.069*	0.549 (5)
C62	0.1039 (5)	0.6592 (7)	0.2597 (6)	0.067 (3)	0.549 (5)
H62A	0.0511	0.6696	0.2064	0.080*	0.549 (5)
H62B	0.1272	0.5836	0.2613	0.080*	0.549 (5)
C63	0.1224 (3)	0.7162 (6)	0.3547 (4)	0.068 (2)	0.549 (5)
H63A	0.1569	0.6757	0.4153	0.082*	0.549 (5)
H63B	0.0809	0.7616	0.3605	0.082*	0.549 (5)
N31	0.48732 (12)	0.6444 (2)	0.37429 (16)	0.0474 (6)	
H31	0.4615 (16)	0.637 (2)	0.314 (2)	0.057*	
S11	0.63319 (4)	0.63305 (6)	0.53535 (5)	0.0480 (2)	
C12	0.56815 (13)	0.6228 (2)	0.40899 (18)	0.0404 (6)	
N13	0.59782 (12)	0.59878 (19)	0.34675 (15)	0.0460 (6)	
C13A	0.68009 (15)	0.5844 (2)	0.39970 (19)	0.0436 (6)	
C14	0.73158 (16)	0.5543 (2)	0.3582 (2)	0.0556 (8)	
H14	0.7124	0.5405	0.2898	0.067*	
C15	0.81099 (17)	0.5452 (3)	0.4194 (2)	0.0581 (8)	
H15	0.8454	0.5232	0.3921	0.070*	
C17	0.79097 (14)	0.5970 (2)	0.5647 (2)	0.0490 (7)	
H17	0.8106	0.6113	0.6330	0.059*	
C17A	0.71007 (14)	0.6035 (2)	0.50220 (19)	0.0406 (6)	
C16A	0.84091 (15)	0.5684 (2)	0.5213 (2)	0.0528 (7)	0.5
O16	0.9220 (6)	0.552 (5)	0.577 (3)	0.065 (7)	0.5
C18	0.9590 (3)	0.5892 (5)	0.6867 (4)	0.0638 (16)	0.5
H18A	1.0156	0.5873	0.7124	0.096*	0.5
H18B	0.9424	0.5369	0.7246	0.096*	0.5
H18C	0.9422	0.6665	0.6922	0.096*	0.5
C16B	0.84091 (15)	0.5684 (2)	0.5213 (2)	0.0528 (7)	0.5
O17	0.9206 (5)	0.576 (5)	0.581 (3)	0.061 (6)	0.5
C19	0.9693 (3)	0.5307 (6)	0.5324 (4)	0.0719 (19)	0.5
H19A	1.0243	0.5366	0.5780	0.108*	0.5
H19B	0.9591	0.5753	0.4733	0.108*	0.5
H19C	0.9561	0.4509	0.5143	0.108*	0.5

### 5-Cyclopropyl-N-(6-methoxybenzo[d]thiazol-2-yl)isoxazole-3-carboxamide (III) Atomic displacement parameters (Å^2^)

**Table:**

	*U*^11^	*U*^22^	*U*^33^	*U*^12^	*U*^13^	*U*^23^
C31B	0.0317 (14)	0.065 (2)	0.0495 (16)	−0.0006 (13)	0.0160 (13)	0.0014 (15)
O31B	0.024 (5)	0.096 (10)	0.0478 (12)	−0.009 (3)	0.0161 (11)	0.0036 (15)
O1B	0.030 (3)	0.072 (4)	0.055 (3)	0.008 (2)	0.0215 (15)	0.0136 (19)
N2B	0.023 (3)	0.068 (5)	0.059 (3)	0.002 (5)	0.019 (3)	0.0044 (17)
C3B	0.0288 (15)	0.053 (4)	0.049 (6)	−0.001 (2)	0.0146 (14)	0.002 (3)
C4B	0.0340 (18)	0.053 (8)	0.062 (9)	0.0033 (16)	0.022 (3)	0.017 (4)
C5B	0.0375 (19)	0.055 (5)	0.051 (5)	−0.002 (2)	0.028 (2)	0.004 (4)
C51	0.039 (4)	0.084 (7)	0.058 (5)	0.002 (5)	0.032 (4)	0.011 (5)
C52	0.033 (4)	0.058 (7)	0.077 (6)	−0.007 (4)	0.026 (4)	0.005 (5)
C53	0.043 (4)	0.058 (5)	0.103 (6)	−0.002 (3)	0.035 (4)	−0.007 (5)
C31A	0.0317 (14)	0.065 (2)	0.0495 (16)	−0.0006 (13)	0.0160 (13)	0.0014 (15)
O31A	0.024 (5)	0.096 (10)	0.0478 (12)	−0.009 (3)	0.0161 (11)	0.0036 (15)
O1A	0.030 (3)	0.072 (4)	0.055 (3)	0.008 (2)	0.0215 (15)	0.0136 (19)
N2A	0.023 (3)	0.068 (5)	0.059 (3)	0.002 (5)	0.019 (3)	0.0044 (17)
C3A	0.0288 (15)	0.053 (4)	0.049 (6)	−0.001 (2)	0.0146 (14)	0.002 (3)
C4A	0.0340 (18)	0.053 (8)	0.062 (9)	0.0033 (16)	0.022 (3)	0.017 (4)
C5A	0.0375 (19)	0.055 (5)	0.051 (5)	−0.002 (2)	0.028 (2)	0.004 (4)
C61	0.028 (3)	0.074 (5)	0.074 (5)	0.007 (3)	0.024 (3)	0.013 (4)
C62	0.035 (4)	0.087 (7)	0.079 (5)	−0.011 (5)	0.025 (4)	−0.018 (6)
C63	0.039 (4)	0.109 (6)	0.067 (4)	0.001 (4)	0.033 (3)	0.000 (4)
N31	0.0273 (11)	0.0672 (16)	0.0421 (12)	0.0001 (11)	0.0096 (10)	−0.0008 (13)
S11	0.0288 (3)	0.0700 (5)	0.0427 (4)	0.0041 (3)	0.0131 (3)	−0.0019 (4)
C12	0.0298 (13)	0.0478 (17)	0.0410 (13)	0.0009 (12)	0.0127 (11)	−0.0001 (13)
N13	0.0367 (12)	0.0544 (15)	0.0441 (12)	0.0048 (10)	0.0146 (10)	−0.0003 (11)
C13A	0.0395 (15)	0.0444 (17)	0.0486 (16)	0.0060 (13)	0.0204 (13)	0.0046 (13)
C14	0.0545 (18)	0.064 (2)	0.0533 (17)	0.0134 (15)	0.0280 (15)	0.0053 (15)
C15	0.0493 (18)	0.067 (2)	0.071 (2)	0.0172 (15)	0.0378 (16)	0.0107 (17)
C17	0.0335 (14)	0.0581 (19)	0.0534 (16)	0.0036 (13)	0.0168 (13)	−0.0011 (14)
C17A	0.0321 (13)	0.0413 (16)	0.0490 (15)	0.0024 (11)	0.0180 (12)	0.0013 (12)
C16A	0.0331 (15)	0.0533 (19)	0.073 (2)	0.0085 (13)	0.0232 (15)	0.0127 (16)
O16	0.038 (6)	0.081 (17)	0.067 (8)	0.012 (4)	0.014 (5)	0.001 (8)
C18	0.039 (3)	0.064 (4)	0.070 (4)	−0.001 (3)	0.006 (3)	0.011 (3)
C16B	0.0331 (15)	0.0533 (19)	0.073 (2)	0.0085 (13)	0.0232 (15)	0.0127 (16)
O17	0.031 (5)	0.084 (18)	0.069 (7)	0.013 (4)	0.022 (5)	−0.008 (7)
C19	0.032 (3)	0.113 (6)	0.074 (4)	0.009 (3)	0.026 (3)	0.003 (4)

### 5-Cyclopropyl-N-(6-methoxybenzo[d]thiazol-2-yl)isoxazole-3-carboxamide (III) Geometric parameters (Å, º)

**Table:**

C31B—O31B	1.227 (7)	C62—H62B	0.9700
C31B—N31	1.352 (3)	C63—H63A	0.9700
C31B—C3B	1.512 (6)	C63—H63B	0.9700
O1B—C5B	1.353 (6)	N31—C12	1.395 (3)
O1B—N2B	1.419 (6)	N31—H31	0.82 (3)
N2B—C3B	1.310 (6)	S11—C12	1.740 (2)
C3B—C4B	1.401 (6)	S11—C17A	1.742 (2)
C4B—C5B	1.350 (7)	C12—N13	1.293 (3)
C4B—H4B	0.9300	N13—C13A	1.405 (3)
C5B—C51	1.474 (7)	C13A—C17A	1.389 (3)
C51—C53	1.477 (7)	C13A—C14	1.391 (3)
C51—C52	1.499 (7)	C14—C15	1.371 (4)
C51—H51	0.9800	C14—H14	0.9300
C52—C53	1.454 (8)	C15—C16A	1.389 (4)
C52—H52A	0.9700	C15—H15	0.9300
C52—H52B	0.9700	C17—C16A	1.383 (3)
C53—H53A	0.9700	C17—C17A	1.395 (3)
C53—H53B	0.9700	C17—H17	0.9300
O1A—C5A	1.357 (5)	C16A—O16	1.393 (8)
O1A—N2A	1.412 (5)	O16—C18	1.52 (4)
N2A—C3A	1.308 (6)	C18—C18^i^	1.840 (10)
C3A—C4A	1.399 (5)	C18—H18A	0.9600
C4A—C5A	1.342 (6)	C18—H18B	0.9600
C4A—H4A	0.9300	C18—H18C	0.9600
C5A—C61	1.473 (6)	O17—C19	1.48 (4)
C61—C63	1.478 (6)	C19—C19^ii^	1.920 (11)
C61—C62	1.503 (7)	C19—H19A	0.9600
C61—H61	0.9800	C19—H19B	0.9600
C62—C63	1.451 (7)	C19—H19C	0.9600
C62—H62A	0.9700		
			
O31B—C31B—N31	119.8 (12)	C62—C63—C61	61.8 (4)
O31B—C31B—C3B	120.3 (12)	C62—C63—H63A	117.6
N31—C31B—C3B	119.0 (14)	C61—C63—H63A	117.6
C5B—O1B—N2B	108.9 (5)	C62—C63—H63B	117.6
C3B—N2B—O1B	104.8 (5)	C61—C63—H63B	117.6
N2B—C3B—C4B	112.0 (5)	H63A—C63—H63B	114.7
N2B—C3B—C31B	119.2 (7)	C31B—N31—C12	124.2 (2)
C4B—C3B—C31B	128.8 (7)	C31B—N31—H31	120.8 (19)
C5B—C4B—C3B	105.5 (6)	C12—N31—H31	114.9 (19)
C5B—C4B—H4B	127.3	C12—S11—C17A	87.99 (11)
C3B—C4B—H4B	127.3	N13—C12—N31	120.4 (2)
C4B—C5B—O1B	108.8 (6)	N13—C12—S11	117.56 (18)
C4B—C5B—C51	133.9 (7)	N31—C12—S11	121.94 (18)
O1B—C5B—C51	117.1 (6)	C12—N13—C13A	109.4 (2)
C5B—C51—C53	123.2 (8)	C17A—C13A—C14	119.4 (2)
C5B—C51—C52	120.0 (7)	C17A—C13A—N13	114.9 (2)
C53—C51—C52	58.5 (4)	C14—C13A—N13	125.8 (2)
C5B—C51—H51	114.6	C15—C14—C13A	119.2 (3)
C53—C51—H51	114.6	C15—C14—H14	120.4
C52—C51—H51	114.6	C13A—C14—H14	120.4
C53—C52—C51	60.0 (4)	C14—C15—C16A	121.1 (3)
C53—C52—H52A	117.8	C14—C15—H15	119.4
C51—C52—H52A	117.8	C16A—C15—H15	119.4
C53—C52—H52B	117.8	C16A—C17—C17A	117.6 (3)
C51—C52—H52B	117.8	C16A—C17—H17	121.2
H52A—C52—H52B	114.9	C17A—C17—H17	121.2
C52—C53—C51	61.5 (4)	C13A—C17A—C17	121.8 (2)
C52—C53—H53A	117.6	C13A—C17A—S11	110.11 (18)
C51—C53—H53A	117.6	C17—C17A—S11	128.1 (2)
C52—C53—H53B	117.6	C17—C16A—C15	120.8 (3)
C51—C53—H53B	117.6	C17—C16A—O16	123 (2)
H53A—C53—H53B	114.7	C15—C16A—O16	116 (2)
C5A—O1A—N2A	108.8 (4)	C16A—O16—C18	118 (3)
C3A—N2A—O1A	103.9 (4)	O16—C18—C18^i^	151.6 (14)
N2A—C3A—C4A	113.4 (5)	O16—C18—H18A	109.5
C5A—C4A—C3A	104.2 (5)	C18^i^—C18—H18A	45.7
C5A—C4A—H4A	127.9	O16—C18—H18B	109.5
C3A—C4A—H4A	127.9	C18^i^—C18—H18B	75.2
C4A—C5A—O1A	109.6 (5)	H18A—C18—H18B	109.5
C4A—C5A—C61	135.3 (6)	O16—C18—H18C	109.5
O1A—C5A—C61	115.1 (5)	C18^i^—C18—H18C	94.3
C5A—C61—C63	119.8 (5)	H18A—C18—H18C	109.5
C5A—C61—C62	120.0 (7)	H18B—C18—H18C	109.5
C63—C61—C62	58.2 (4)	O17—C19—C19^ii^	178.6 (18)
C5A—C61—H61	115.6	O17—C19—H19A	109.5
C63—C61—H61	115.6	C19^ii^—C19—H19A	71.7
C62—C61—H61	115.6	O17—C19—H19B	109.5
C63—C62—C61	60.0 (3)	C19^ii^—C19—H19B	70.7
C63—C62—H62A	117.8	H19A—C19—H19B	109.5
C61—C62—H62A	117.8	O17—C19—H19C	109.5
C63—C62—H62B	117.8	C19^ii^—C19—H19C	69.2
C61—C62—H62B	117.8	H19A—C19—H19C	109.5
H62A—C62—H62B	114.9	H19B—C19—H19C	109.5
			
C5B—O1B—N2B—C3B	0 (4)	C5A—C61—C62—C63	108.5 (7)
O1B—N2B—C3B—C4B	1 (6)	C5A—C61—C63—C62	−108.9 (8)
O1B—N2B—C3B—C31B	179 (4)	O31B—C31B—N31—C12	4.6 (15)
O31B—C31B—C3B—N2B	132 (4)	C3B—C31B—N31—C12	174 (2)
N31—C31B—C3B—N2B	−37 (6)	C31B—N31—C12—N13	179.8 (3)
O31B—C31B—C3B—C4B	−49 (6)	C31B—N31—C12—S11	−3.1 (4)
N31—C31B—C3B—C4B	141 (5)	C17A—S11—C12—N13	0.7 (2)
N2B—C3B—C4B—C5B	−2 (6)	C17A—S11—C12—N31	−176.5 (2)
C31B—C3B—C4B—C5B	180 (4)	N31—C12—N13—C13A	178.1 (2)
C3B—C4B—C5B—O1B	2 (5)	S11—C12—N13—C13A	0.9 (3)
C3B—C4B—C5B—C51	176 (3)	C12—N13—C13A—C17A	−2.6 (3)
N2B—O1B—C5B—C4B	−1 (3)	C12—N13—C13A—C14	177.8 (3)
N2B—O1B—C5B—C51	−176.4 (18)	C17A—C13A—C14—C15	−0.6 (4)
C4B—C5B—C51—C53	155 (3)	N13—C13A—C14—C15	178.9 (2)
O1B—C5B—C51—C53	−31.4 (18)	C13A—C14—C15—C16A	−1.6 (4)
C4B—C5B—C51—C52	−135 (3)	C14—C13A—C17A—C17	2.0 (4)
O1B—C5B—C51—C52	38.5 (18)	N13—C13A—C17A—C17	−177.5 (2)
C5B—C51—C52—C53	−112.8 (9)	C14—C13A—C17A—S11	−177.3 (2)
C5B—C51—C53—C52	107.4 (10)	N13—C13A—C17A—S11	3.2 (3)
C5A—O1A—N2A—C3A	0 (3)	C16A—C17—C17A—C13A	−1.2 (4)
O1A—N2A—C3A—C4A	−2 (5)	C16A—C17—C17A—S11	177.9 (2)
N2A—C3A—C4A—C5A	3 (5)	C12—S11—C17A—C13A	−2.1 (2)
C3A—C4A—C5A—O1A	−2 (4)	C12—S11—C17A—C17	178.7 (3)
C3A—C4A—C5A—C61	178 (2)	C17A—C17—C16A—C15	−1.1 (4)
N2A—O1A—C5A—C4A	2 (2)	C17A—C17—C16A—O16	−176 (3)
N2A—O1A—C5A—C61	−178.5 (14)	C14—C15—C16A—C17	2.5 (5)
C4A—C5A—C61—C63	6 (3)	C14—C15—C16A—O16	178 (3)
O1A—C5A—C61—C63	−173.6 (9)	C17—C16A—O16—C18	−12 (5)
C4A—C5A—C61—C62	−62 (3)	C15—C16A—O16—C18	173 (3)
O1A—C5A—C61—C62	118.1 (10)	C16A—O16—C18—C18^i^	165.7 (9)

Symmetry codes: (i) −*x*+2, *y*, −*z*+3/2; (ii) −*x*+2, −*y*+1, −*z*+1.

### 5-Cyclopropyl-N-(6-methoxybenzo[d]thiazol-2-yl)isoxazole-3-carboxamide (III) Hydrogen-bond geometry (Å, º)

**Table:**

*D*—H···*A*	*D*—H	H···*A*	*D*···*A*	*D*—H···*A*
N31—H31···N13^iii^	0.82 (3)	2.19 (3)	3.003 (3)	173 (2)
C17—H17···O1*A*^iv^	0.93	2.51	3.293 (7)	142
C17—H17···N2*A*^iv^	0.93	2.55	3.440 (19)	160
C63—H63*B*···O31*A*^v^	0.97	2.58	3.440 (18)	148

Symmetry codes: (iii) −*x*+1, *y*, −*z*+1/2; (iv) *x*+1/2, −*y*+3/2, *z*+1/2; (v) −*x*+1/2, −*y*+3/2, −*z*+1.
